# A Photoredox Reaction for the Selective Modification of 5-Carboxycytosine in DNA

**DOI:** 10.1021/jacs.2c12558

**Published:** 2023-05-04

**Authors:** Benjamin J. Mortishire-Smith, Sidney M. Becker, Angela Simeone, Larry Melidis, Shankar Balasubramanian

**Affiliations:** † Yusuf Hamied Department of Chemistry, University of Cambridge, Cambridge, CB2 1EW, U.K.; ‡ Cancer Research UK Cambridge Institute, Li Ka Shing Centre, University of Cambridge, Cambridge, CB2 0RE, U.K.; § School of Clinical Medicine, University of Cambridge, Cambridge, CB2 0SP, United Kingdom

## Abstract

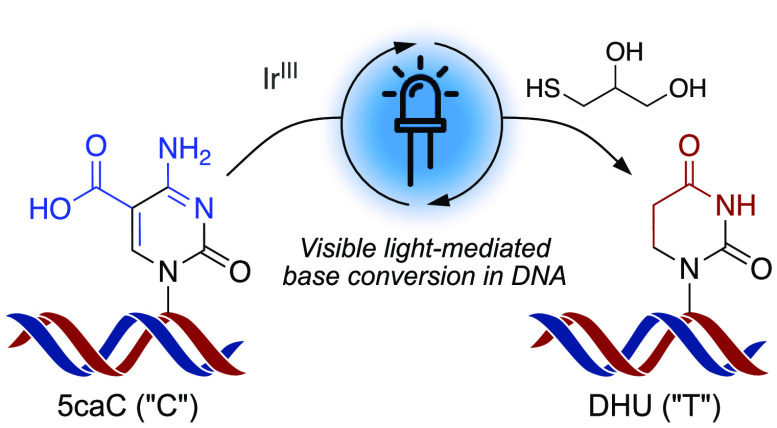

Covalent epigenetic modifications contribute to the regulation of important cellular processes during development and differentiation, and changes in their genomic distribution and frequency are linked to the emergence of genetic disease states. Chemical and enzymatic methods that selectively target the orthogonal chemical functionality of epigenetic markers are central to the study of their distribution and function, and considerable research effort has been focused on the development of nondestructive sequencing approaches which preserve valuable DNA samples. Photoredox catalysis enables transformations with tunable chemoselectivity under mild, biocompatible reaction conditions. We report the reductive decarboxylation of 5-carboxycytosine via a novel iridium-based treatment, which represents the first application of visible-light photochemistry to epigenetic sequencing via direct base conversion. We propose that the reaction involves an oxidative quenching cycle beginning with single-electron reduction of the nucleobase by the photocatalyst, followed by hydrogen atom transfer from a thiol. The saturation of the C5–C6 backbone permits decarboxylation of the nonaromatic intermediate, and hydrolysis of the N4-amine constitutes a conversion from a cytosine derivative to a T-like base. This conversion demonstrates selectivity for 5-carboxycytosine over other canonical or modified nucleoside monomers, and is thereby applied to the sequencing of 5-carboxycytosine within modified oligonucleotides. The photochemistry explored in this study can also be used in conjunction with enzymatic oxidation by TET to profile 5-methylcytosine at single-base resolution. Compared to other base-conversion treatments, the rapid photochemical reaction takes place within minutes, which could provide advantages for high-throughput detection and diagnostic applications.

## Introduction

DNA-encoded information is fundamental to the development and function of living things. The DNA nucleobases which comprise the canonical four-letter code can be modified *in vivo* through the addition of covalent functional groups. These constitute the epigenetic code, a secondary layer of information which dynamically and reversibly modulates gene expression via molecular interactions with DNA-binding proteins, without altering the primary DNA sequence.^1^ 5-Methylcytosine (5mC) is the most abundant modified base in the mammalian genome, representing up to 5% of total cytosine sites, and is important to the biology of normal function and disease.^2^ In particular, cytosine methylation regulates key processes such as cell differentiation, X-inactivation, and genomic imprinting, while aberrant methylation patterns are linked to the emergence of cancer states.^3,4^ Ten-eleven translocation (TET) dioxygenases can successively oxidize 5mC to 5-hydroxymethylcytosine (5hmC), 5-formylcytosine (5fC), and 5-carboxycytosine (5caC).^5^ 5fC and 5caC are targeted for base excision repair (BER) by thymine DNA glycosylase (TDG), which provides a pathway for demethylation of 5mC. The oxidized cytosine bases can also exist at stable levels within DNA,^6,7^ are enriched within regulatory enhancer and promoter regions,^8,9^ and may constitute epigenetic marks in their own right with distinct regulatory roles. For example, potential readers of 5fC and 5caC have been identified,^10,11^ and these modifications have been observed to transiently inhibit transcription by RNA Pol II.^12^ However, 5caC in particular remains poorly understood, and the relatively low genomic abundances of 5fC and 5caC (fewer than one base in 10^5^ and 10^6^, respectively) make it difficult to investigate their functions. Efficient chemical methodologies for the labeling, detection, imaging, and editing of 5caC and other modified bases are required to address this challenge and to better understand their roles in regulation, development, and disease.^13^

**Figure 1:**
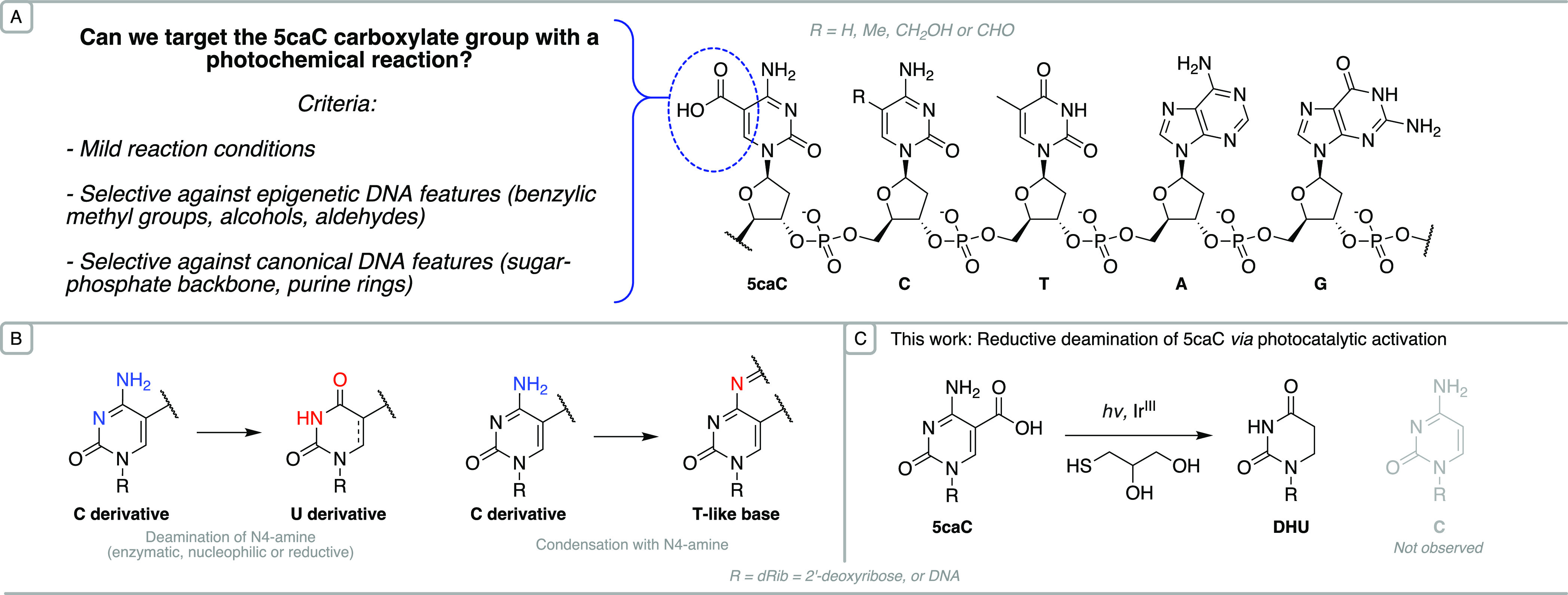
Context for the selective photoredox modification of 5caC. (A) Structure of canonical bases and modified cytosines within a DNA strand. (B) The base pairing preferences of cytosine derivatives are altered selectively upon deamination or condensation. (C) A new reaction for the formation of 5,6-dihydrouridine (DHU) from 5caC, involving photochemical activation in the presence of a thiol to achieve reduction and decarboxylation under nonforcing conditions.

The natural epigenetic modifications expand the spectrum of orthogonal chemical functionalities within a DNA molecule, thereby offering targets for the development of chemoselective reactions for the selective manipulation of base derivatives (Figure 1A). Chemistries that alter the hydrogen-bonding pattern of the Watson–Crick face of bases are of particular interest, as they allow epigenetic modifications to be profiled at single-nucleotide resolution.

The most widely used example of this approach is bisulfite treatment, which enables the detection of 5mC via the selective deamination of cytosine to uracil.^14^ When paired with sequencing, unmodified cytosine can subsequently be distinguished from 5-methylcytosine. This technique has also been incorporated into a range of methodologies which can differentiate between the remaining oxidized cytosines.^15^ However, the bisulfite treatment can degrade more than 95% of input material^16^ and reduces DNA sequence complexity to a three-letter alphabet (via deamination of unmodified cytosines), hindering bioinformatic analysis. Therefore, bisulfite-free approaches which selectively alter base identity while preserving the structure and complexity of DNA fragments are therefore of interest. Such strategies have included deamination of 5mC and 5hmC by AID/APOBEC enzymes,^17,18^ reduction and deamination of 5fC/5caC by pyridine borane,^19^ and Friedländer condensation of 5fC with indandione or malononitrile^20^ (Figure 1B).

Herein, we describe a novel photochemical reaction that is specific to the noncanonical base 5caC and demonstrate its application to the detection of 5caC and 5mC in oligonucleotide and DNA contexts. In addition to this unique chemoselectivity, this is the first example of photoredox chemistry enabling epigenetic sequencing of a nucleotide at single-base resolution.

## Results and Discussion

### Rationale for Reaction Design

Visible-light-mediated photoredox chemistry can catalyze rapid transformations with high selectivity under mild conditions, including in aqueous solvents at physiological pH.^21^ It is therefore well-suited to the manipulation of complex biomolecules, and methods for the targeted labeling of proteins have exemplified the utility of single-electron chemistry in this context.^22,23^ Indeed, we recently developed a selective photochemical C–H functionalization of an adenine modification in DNA.^24^ Since photocatalytic transformations of biomolecules are viable in aqueous conditions, we considered the unique functionality of 5caC in DNA to be a promising target for a similar chemistry (Figure 1A). Single-electron oxidation of a carboxylate group by a photoactivated species can enable rapid and favorable extrusion of CO_2_ to generate a carbon-centered radical.^25^ Decarboxylation of aliphatic substrates produces a relatively stable sp^3^ radical, which can either abstract a hydrogen atom or undergo coupling with a range of electrophiles such as Michael acceptors. In comparison, the direct decarboxylation of aromatic compounds is difficult; few examples of these transformations have been reported and rely on nonbiocompatible prior derivatizations via silver salts^26,27^ or hypobromite intermediates.^28^ Although an aromatic carboxylate group itself may undergo single-electron exchange with a suitable catalyst, subsequent decarboxylation to an unfavorable sp^2^ radical takes place at a slower rate than regeneration of the starting material via back-electron or hydrogen atom transfer.^23^

Canonical DNA nucleosides possess single-electron standard reduction potentials ranging from below −2.74 V to −2.12 V vs NHE.^29^ However, due to electron withdrawal by the carboxylate group, a single electron reduction of the nucleobase was expected to be more accessible for 5-carboxycytosine than for unmodified cytosine (*E* = −2.21 V vs NHE), providing a potential window for a chemoselective reaction. Thus, we sought to explore the reactivity of 5caC in combination with a photoredox catalyst and a thiol hydrogen atom donor (Figure 1C).

### Identification of Novel 5caC Reactivity

**Figure 2:**
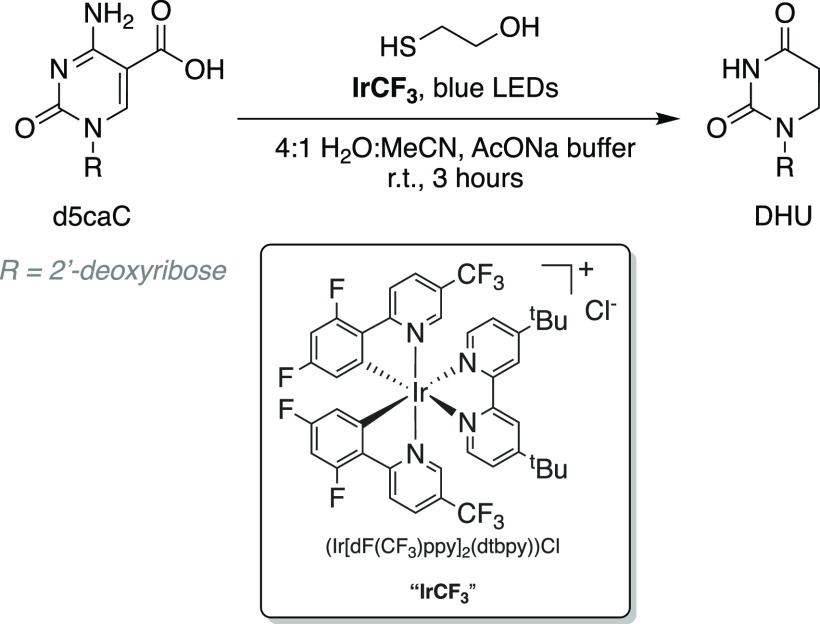
Photocatalytic conversion of 5-carboxycytidine to 5,6-dihydrouracil.

2′-Deoxy-5-carboxycytosine (d5caC) was synthesized for use in reaction screening based on reported procedures.^30^ 2-Mercaptoethanol was initially selected as the thiol for its high aqueous solubility. A partial consumption of 5caC (7.4% reduction in UV–vis absorption by LC-MS, Figure S1) resulted from a 3 h incubation using photocatalyst [Ir(dF(CF_3_)ppy)_2_(dtbbpy)]Cl (0.25 M thiol, 100 mM/pH 4.5 sodium acetate buffer). Reaction efficiency was moderately improved at higher thiol concentrations (31.2% consumption using 1.0 M thiol, Figure S2). The further addition of a small amount of organic cosolvent (20% v/v acetonitrile) serving to increase catalyst solubility resulted in near-quantitative reaction within 3 h (>98%; Figure S3). The observation of a single new nucleoside species with a molecular weight change of −41 Da and weak 260 nm absorbance (Figure S4) suggested the formation of 2′-deoxy-5,6-dihydrouridine (DHU). This result was confirmed upon isolation of the product and characterization using HRMS and NMR spectroscopy, and represents conversion of the C-derivative to a T-like base (Figure 2).

Other photocatalysts displayed poor solubility and limited reactivity in the aqueous solvent system; thus, the chemistry was explored further using photocatalyst [Ir(dF(CF_3_)ppy)_2_(dtbbpy)]Cl and 2-mercaptoethanol. No deglycoslyation of the nucleoside was detected by LC-MS analysis, and notably, nitrogen sparging to remove oxygen was not necessary for the reaction to proceed smoothly. Alternative cosolvents were also found to be suitable for the reaction (Table S1).

In order to evaluate the chemoselectivity of the reaction, other canonical and modified bases were treated under identical conditions. LC-MS analysis after illumination for 3 h detected no change in mass or the intensity of UV–vis absorbance for 2′-deoxythymidine (dT) or 2′-deoxyadenosine (dA) (Table 1). The deamination and base-pairing change induced by the photoreaction demonstrated excellent selectivity for 5-carboxycytosine over unmodified cytosine and its other derivatives.

**Table 1:** Reactivity of Canonical and Modified Nucleosides under the Photochemical Conditions

Entry	Starting material	Change in base-pairing properties^a^ (%)
1	dA	n.d.
2	dG	n.d.
3	dT	n.d.
4	dC	2.3
5	d5mC	2.3
6	d5hmC	<1
7	d5fC	<1
8	d5caC	99

a Nucleoside concentrations calculated based on ratio of integrated UV–vis absorbance (260 nm) before and after 3 h incubations, relative to an internal dA standard. Reactions were repeated twice. n.d.: not detected (Figures S5–12, Table S2). Entries 5–8 represent the 2′-deoxynucleosides of 5-methylcytidine (d5mC), 5-hydroxymethylcytidine (d5hmC), 5-formylcytidine (d5fC), and 5-carboxycytidine (d5caC).

### Mechanistic Insights

The conversion of 5caC to DHU takes place via a combination of C5–C6 reduction, C5 decarboxylation, and N4 deamination. Control experiments demonstrated that the reaction does not occur in the absence of the photocatalyst, light, or 2-mercaptoethanol. A substrate analogue, 5-methylcarboxy-2′-deoxycytidine (d5mecaC), underwent C5–C6 reduction without ester cleavage (Figure S13), indicating that the reduction is an independent dearomatizing step that permits subsequent decarboxylation of 5caC. Stern–Volmer analysis in the reaction solvent in the absence of 2-mercaptoethanol determined that the d5caC nucleobase is a quencher of the excited iridium photocatalyst, and a similar quenching effect observed with d5mecaC ruled out interactions with the carboxylic acid moiety (Figures S14–15). On the other hand, catalyst fluorescence was not detectably decreased by dC (Figure S16), which is considerably less reactive in the photoreaction than d5caC. The quantitative correlation between quenching interactions and the reactivity of substrates suggests that the reduction is initiated by an interaction between the nucleobase and excited photocatalyst, which is independent of the thiol and results from electronic properties shared between d5caC and d5mecaC.

A computational investigation provided insight into the chemoselectivity between 5-carboxycytosine/5-methylcarboxycytosine and unmodified cytosine nucleobases. DFT calculations (see Supporting Information) predicted electron transfer to be more accessible in the case of 5-carboxycytosine and 5-methylcarboxycytosine compared to cytosine (Table 2, Table S3). Single-electron reduction potentials of nucleobases were measured experimentally using cyclic voltammetry and correlated with the predicted values. Overall, a reduction of 5-carboxycytosine (or 5-methylcarboxycytosine) is approximately 0.5 V more accessible (less negative) than that of unmodified cytosine, and is qualitatively separate from the range reported for other canonical nucleobases.^29^ DFT calculations also indicated the C6 position of the radical anions to be most susceptible to further reaction, in line with experimental observations (Figures S18–19).

**Table 2:** Experimental and Calculated Reduction Potentials (V vs NHE)

	Experimental value^a^	Calculated value^b^
dC	–2.25	–2.52
d5caC	–1.71	–1.76
d5mecaC	–1.78	–1.92

a Reduction potentials of 2′-deoxyribonucleosides (N/N^•–^) were measured by cyclic voltammetry in dry, degassed DMF and are reported as half-peak potentials relative to Fc^+^/Fc.

b 1-Methyl-pyrimidine nucleobases were modeled to obtain calculated values.

**Figure 3:**
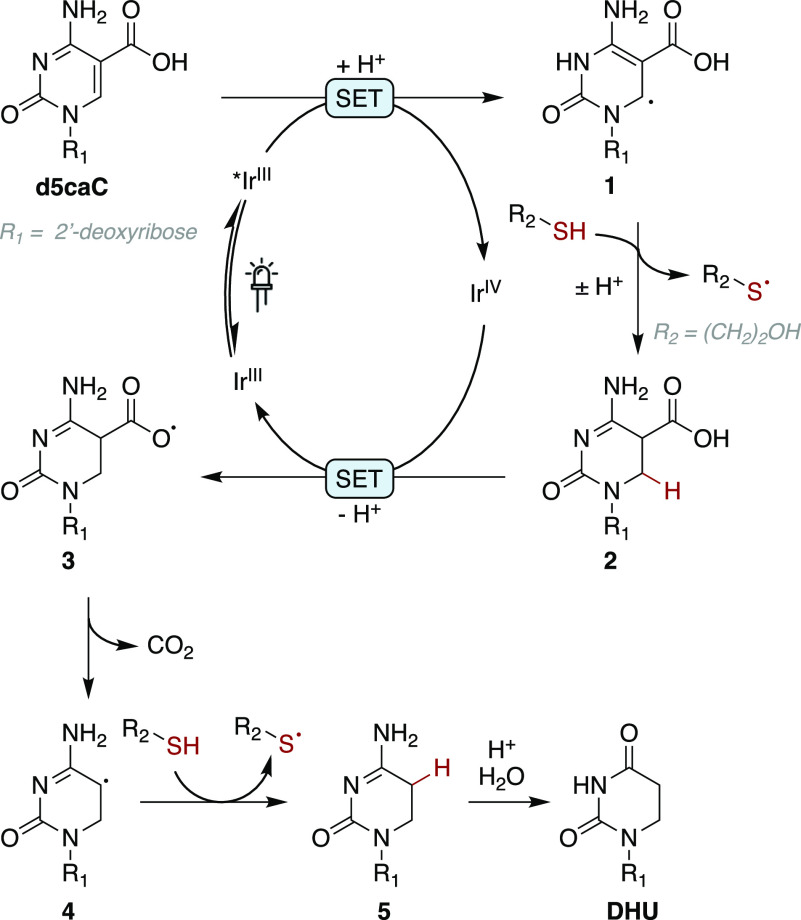
Proposed mechanism for the photochemical conversion.

On the basis of these observations, we propose the following pathway as a plausible mechanism for the reaction (Figure 3).

The iridium photocatalyst is converted to a single-electron reductant upon absorption of blue light and intersystem crossing to the triplet-excited state.^31,32^ Delocalized radical **1** is formed by electron transfer from the catalyst to the unsaturated carbonyl, representing an oxidative quenching cycle; in aqueous solution with 2-mercaptoethanol, this reduction may be further facilitated at pH 4.5 by protonation at the N3 nitrogen (p*K*_a_ = 4.2).^33^ Hydrogen atom transfer (HAT) to this C6 carbon radical from 2-mercaptoethanol (calculated S–H BDE = 84.9 kcal mol^–1^) results in reduction to nucleoside **2**; a high concentration of the thiol is necessary to compete with back electron transfer and drive the reaction forward. Faster nucleoside reaction rates were observed with electron-deficient thiols cysteamine and thioacetic acid (Figure S20), which may be due to their lower S–H BDEs (83.5 (with protonated amine) and 83.6 kcal mol^–1^ respectively) or improved polarity-matched HAT^34^ with nucleophilic radical **1** (Table S4).

The catalytic cycle is closed upon electron transfer to the oxidized iridium complex and formation of carboxyl radical **3**, which undergoes extrusion of CO_2_ (**4**) followed by hydrogen atom transfer to complete the reduction to intermediate **5**; alternatively, an ionic decarboxylation of nucleoside **2** may be stabilized by the conjugated π-system. Finally, N4-deamination of intermediate **5** to form 2′-deoxy-5,6-dihydrouridine is spontaneous under the mildly acidic reaction conditions. The disulfide of 2-mercaptoethanol was identified by LC-MS as a byproduct of the reaction, and is likely to result from the coupling of thiyl radicals produced during hydrogen atom transfer. Although the conjugate addition of 2-mercaptoethanol has been reported to catalyze an alternative decarboxylation of d5caC,^35^ no conjugate adduct or accumulation of unreactive dC is observed in reaction mixtures; thus, any such side reactions are not competitive with the photocatalytic pathway.

### Application of Reaction to DNA Oligonucleotides

**Figure 4:**
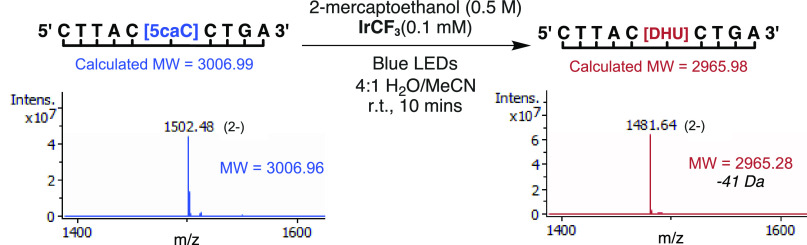
ESI-MS characterization of the conversion of a modified DNA 10mer.

Encouraged by the selectivity observed in nucleoside reactions, we next explored whether the chemistry could be applied to DNA oligomers. The relatively complex structure of DNA poses additional chemoselectivity challenges: the DNA backbone contains phosphate linkages and a variety of C–H bonds which provide opportunities for off-target reactivity or degradation. The reactivities of the DNA nucleobases inside a strand are also modulated by electronic π-stacking and steric effects. Furthermore, nondestructive chemical conditions are desirable as the small quantities of DNA available from some biological samples can be a limiting factor in sequencing applications. A single-stranded 10-mer containing one 5caC base was incubated at lower reagent concentrations (0.1 mM photocatalyst, 0.5 M thiol, Figure S21). Gratifyingly, after illumination for just 10 min, this treatment induced a molecular weight reduction of 41 Da, representing conversion of 5caC to DHU within the oligonucleotide strand (Figure 4). The accompanying peak-to-single-peak shift in the mass spectra and UV chromatograms indicated qualitative conversion without detectable off-target reactivity, while the UV–vis absorbance showed no significant change in oligomer concentration. These results were reproducible using other oligomer sequences covering the remaining three CpN dinucleotide contexts.

### Detection of 5caC via Photochemistry and Next-Generation Sequencing

We explored the feasibility of detecting 5caC using the photochemical treatment in conjunction with next-generation sequencing in two systems of increasing complexity: (A) a synthetic 74-base oligonucleotide containing one 5caC modification, and (B) bacteriophage-λ genomic DNA in which 5caC modifications were artificially introduced at defined positions. As uracil-tolerant DNA polymerases are able to efficiently bypass DHU while incorporating a complementary adenosine base, converted 5caC positions are detected as C-to-T mutations following DNA replication.^36^

**Figure 5:**
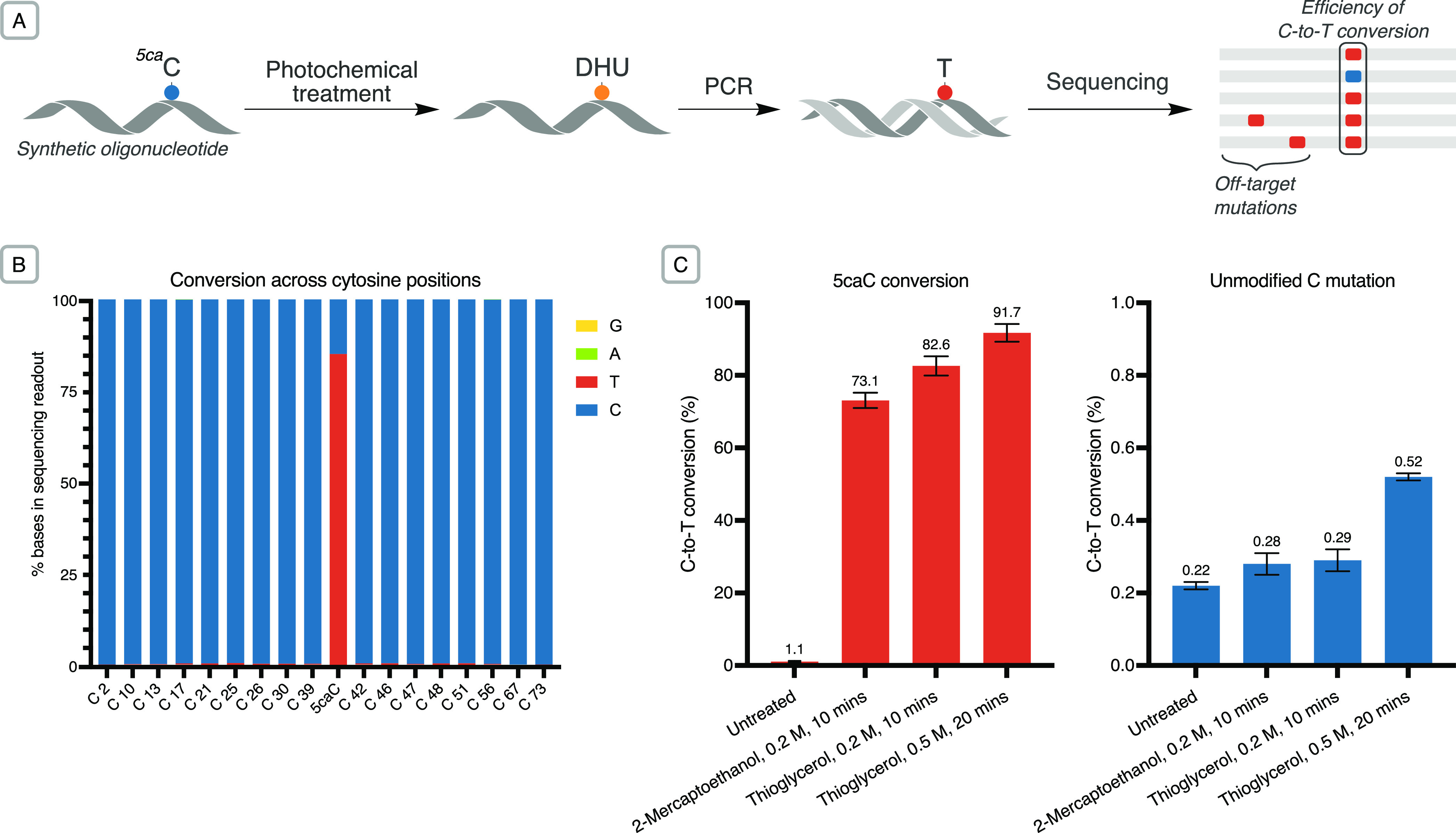
(A) The photochemical conversion was applied to single-stranded DNA oligonucleotides and evaluated via next-generation sequencing. (B) Stacked barplot of the C-to-T conversion in sequencing readout of different cytosine bases across a modified oligomer (0.2 M thioglycerol, 0.1 mM [Ir(dF(CF_3_)ppy)_2_(dtbbpy)]Cl, 10 min illumination; base 41 = 5caC). (C) Barplot of mean conversion rates (±std deviation, *n* = 3) of 5caC (position 41) and unmodified cytosine to T, detected in sequencing following various chemical treatments.

In the first system (Figure 5A), the photochemical conversion efficiency of 5caC to DHU was detected as the fraction of aligned reads containing T at the target base (position 41, Figure 5B) following PCR amplification and sequencing of each sample. This workflow was used to explore reaction conditions for sequencing performance. Thioglycerol was identified as the thiol providing the highest conversion of 5caC and selectivity over canonical bases in the oligonucleotide context (Figures S22–23). An average C-to-T conversion efficiency of 82.6% was observed at the 5caC position following treatments for 10 min using 0.2 M thiol (Figure 5C). The average conversion of unmodified cytosines throughout the oligonucleotide was 0.29% (versus 0.22% in untreated controls).

**Figure 6:**
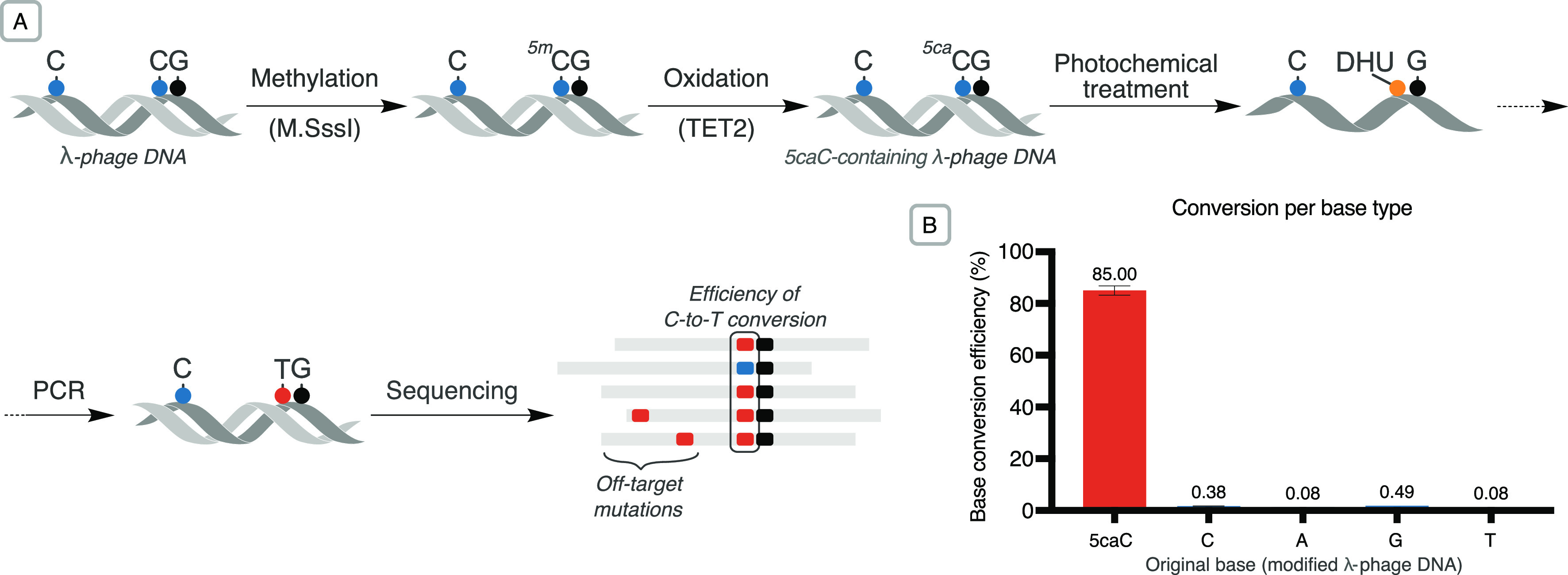
(A) Modified λ-phage DNA was subjected to various photochemical conditions and the results evaluated via next-generation sequencing. (B) Barplot of mean base conversion rates (±std deviation, *n* = 2) resulting from photochemical treatment of the modified genome (0.5 M thioglycerol, 0.1 mM photocatalyst, 20 min illumination followed by 2 h hydrolysis at 37 °C; Table S6, entry 8). The rate of on-target 5caC-to-T transitions observed at CpG sites during base calling was corrected to account for unmethylated and unoxidized bases.

Second, we applied the photochemistry to a small modified genome (bacteriophage-λ, 48.5 kb). 5caC bases were installed enzymatically at CpG dinucleotides (a C base followed by a G) via M.SssI methylation followed by oxidation with TET2, which is able to oxidize 5mC to 5caC (Figure 6A).^18^ The proportion of 5caC modifications introduced at CpG dinucleotides was determined analytically via digestion and LC-MS analysis (Table S5). As single-stranded DNA is required for this chemical conversion, oxidized λ-DNA samples were denatured at 95 °C immediately prior to the photochemical treatment. Sequencing reads from treated DNA were processed and aligned to the reference λ genome. The efficiency of the 5caC conversion step was calculated as the percentage of on-target C-to-T transitions in aligned sequencing reads relative to the 5caC content of CpG dinucleotides in the input DNA. Following further exploration of photochemical conditions via this workflow, a C-to-T conversion efficiency of up to 85.0% of 5caC bases was achieved in sequencing, versus 0.38% off-target conversion of unmodified C (Figure 6B, Table S6). These results demonstrate that the photochemical reaction can be successfully applied to the detection of 5-methylcytosine in the context of a genomic DNA workflow.

## Conclusions

We have described, to the best of our knowledge, the first direct chemical conversion of the identity of a DNA base via photoredox chemistry. The reaction implements a novel radical reduction and decarboxylation of an aromatic compound, and is selective for 5-carboxycytosine over other canonical and modified DNA bases. Applications of the bisulfite-free chemistry include a single-step detection of 5-carboxycytosine, and also the two-step sequencing of 5-methylcytosine. We envisage considerable potential for further applications of photoredox chemistry in the chemical manipulation of nucleic acids for detection and sequencing.

## Supporting Information Available

Materials and methods, experimental procedures, ^1^H and ^13^C NMR spectra, LC-MS data, HR-MS data, Stern–Volmer analysis, cyclic voltammetry, DFT and computational analysis, sequencing data (PDF)
