# Supplementary material for: A Photoredox Reaction for the Selective Modification of 5-Carboxycytosine in DNA
Source: J Am Chem Soc. 2023 May 4;145(19):10505–11. doi: 10.1021/jacs.2c12558 (PMC10197125; doi:10.1021/jacs.2c12558)
Supplement: Supplementary file 1 — ja2c12558_si_001.pdf [file ja2c12558_si_001.pdf]

Supplementary information

A photoredox reaction for the selective modification of 5-carboxycytosine in DNA

Benjamin J. Mortishire-Smith[1], Sidney M. Becker[1], Angela Simeone[1,2], Larry Melidis[1,2], Shankar Balasubramanian[1,2,3]*

[1]Yusuf Hamied Department of Chemistry, University of Cambridge, Cambridge, CB2 1EW, United Kingdom

[2]Cancer Research UK Cambridge Institute, University of Cambridge, Cambridge, CB2 0RE, United Kingdom

[3]School of Clinical Medicine, University of Cambridge, Cambridge, CB2 0SP, United Kingdom

*: Corresponding author: sb10031@cam.ac.uk

Containing materials and experimental methods, general procedures, Supplementary Methods A to D, computational methods, Figures S1 to S26, Tables S1 to S6, NMR spectra. Next-generation sequencing data are available at DOI 10.6084/m9.figshare.19174544

Table of Contents

1 – Materials and methods ***3***

2 – General procedures ***4***

2A – Synthetic methodology **4**
d5caC nucleoside synthesis 4
d5mecaC nucleoside synthesis 4
Catalyst preparation 4

2B – Photoreaction procedures **5**
Supplementary method A: Nucleoside photoreactions 5
Supplementary method B: Oligonucleotide photoreactions 5

2C – Next-Generation DNA sequencing procedures **6**
Supplementary method C: Synthetic oligonucleotides 6
Supplementary method D: λ-phage genomic DNA 7

3 – Experimental data ***9***

3A – Exploration of d5caC nucleoside reactivity **9**
Key LC-MS chromatograms 9
Isolation & characterisation of DHU product 12
Further LC-MS chromatograms: Reaction development 13

3B – Mechanistic study **17**
Substrate analogue 17
Stern-Volmer analysis 18
Computational study 20
Cyclic voltammetry 20
Thiol rate comparisons, S-H bond dissociation energies and charge calculations 22

3C – Reactions on model oligonucleotides **23**

3D – Next-generation sequencing **24**
Synthetic oligonucleotides 24
λ-Phage DNA 27

3E – NMR spectra **28**

References ***31***

1 – Materials and methods

All solvents and reagents were used as supplied from commercial sources Sigma-Aldrich, Alfa Aesar, or Fisher Scientific, without further purification. Ir(dF(CF3)ppy)2(dtbbpy)]PF6 was obtained from Manchester Organics. 2'-Deoxy-5,6-dihydrouridine (DHU) was obtained from Santa Cruz Biotechnology. All reactions were performed at room temperature unless otherwise stated. Solvent removal was accomplished with a Genevac EZ-2 Elite evaporator or a ScanVac CoolSafe lyophiliser.

Flash chromatography was carried out on a Teledyne ISCO Combiflash NextGen 300 with RediSep High Performance Gold Silica columns (4 g to 60 g), using a flow rate of 30 mL min^{-1} at room temperature with in-house distilled solvents. High performance liquid chromatography (HPLC) was carried out on an Interchim Puriflash 4250 MS using an Interchim Puriflash Prep-LC C18 column (150 x 21.2 mm, 15 µm) and gradient elution with water/acetonitrile (MeCN) containing 0.1 % v/v TFA at a flow rate of 15 mL min-1.

Photoreactions were illuminated using a PhotoRedOx Box equipped with an EvoluChem 450DX light source (HepatoChem, 450 nm, 30 W).

Synthetic and screening reactions were monitored with tandem liquid chromatography-mass spectrometry (LC-MS) on a Bruker amaZon X Ion Trap MS, with a Supelcosil LC-18-S nucleoside column (Sigma-Aldrich, 25 cm x 4.6 mm, 5 µm) or an Acquity Premier BEH C18 oligonucleotide column (Waters, 2.1 x 50mm, 1.7 µm). UV chromatograms are reported from 260 nm absorbance, unless otherwise specified.

Proton and carbon nuclear magnetic resonance (1H NMR and 13C NMR) spectra were acquired with either a Bruker Avance III HD (400 MHz) spectrometer at ambient temperature or Bruker Avance III DCH Cryoprobe (500 MHz), using deuterated solvents as indicated. Chemical shifts (δ) are reported in parts per million (ppm) relative to the residual solvent, and coupling constants (J) are reported in hertz (Hz). Multiplicity is reported using combinations of the following abbreviations: s = singlet, d = doublet, t = triplet, m = multiplet/overlapping peaks, br = broad. Analysis of NMR spectra was performed using MestReNova software. High resolution mass spectra (HRMS) for small molecules were recorded on a Waters LCT Premier or ThermoFinnigan Orbitrap Classic mass spectrometer. Stern-Volmer analysis was performed using a Cary Eclipse fluorescence spectrophotometer. Cyclic voltammetry was carried out with an Autolab PGSTAT204 potentiostat and data analysis was performed using Nova software.

Solid phase synthesis of 10-mer oligonucleotides was performed in-house on an ABI394 Synthesizer using standard phosphoramidite chemistry, with reagents supplied by Biosearch Technologies. d5caC phosphoramidite was obtained from Glen Research. Synthesised oligonucleotides were purified by reversed-phase HPLC using a linear gradient of acetonitrile in 0.1M triethylamine acetate on a Clarity 5 µm oligo-RP column (Phenomenex). Custom PCR primers were obtained from Sigma-Aldrich. Modified 74-mer oligonucleotides were synthesised by ATDBio with HPLC purification and used as supplied.

Molecular biology kits and enzymes were obtained from Zymo Research, Thermo Fisher Scientific, Qiagen, Roche, and NEB. Library preparation reagents including enzymes and index primers were supplied by NEB, Roche and IDT. Unmethylated λ-phage DNA was supplied by ProMega.

DNA fragmentation was carried out using a Covaris M220 ultrasonicator. Library distributions were analysed using an Agilent 2200 TapeStation with HS D1000 ScreenTapes & reagents. Next-generation sequencing was performed on an Illumina MiSeq System using MiSeq Reagent nano v2 or v3 kits.

2 – General procedures

2A – Synthetic methodology

d5caC nucleoside synthesis

2'-Deoxy-5-carboxycytidine was synthesised following reported procedures.[1,2] The obtained nucleoside exhibited spectral properties in agreement with the literature values: ^{1}H NMR (400 MHz, D_2O): δ 8.51 (s, 1H), 6.16 (t, J = 6.4 Hz, 1H), 4.37 (dt, J = 6.5, 4.2 Hz, 1H), 4.00 (dt, J = 5.1, 3.7 Hz, 1H), 3.78 (dd, J = 12.5, 3.5 Hz, 1H), 3.69 (dd, J = 12.5, 5.1 Hz, 1H), 3.49 (d, J = 10.7, <1H), 2.40 (ddd, J = 14.2, 6.5, 4.3 Hz, 1H), 2.25 (dt, J = 14.2, 6.5 Hz, 1H); ^{13}C NMR (101 MHz, D_2O): δ 169.58, 162.60, 153.70, 147.08, 102.36, 87.00, 86.64, 70.29, 61.04, 39.57; HRMS: $[M+H]^+$ for $[C_{10}H_{14}N_3O_6]^+$ calculated 272.0877, found 272.0669.

d5mecaC nucleoside synthesis

An intermediate in the synthesis of d5caC was separately deprotected without ester hydrolysis to provide the methylated nucleoside. TBS-protected 2'-deoxy-5-carboxymethylcytidine (0.498 g, 0.973 mmol) was dissolved in THF (5 mL) and TBAF (1.0 M in THF, 2.92 mL, 3 eq.) was added dropwise. The reaction was stirred at room temperature for 1 hour and purified by flash column chromatography (0 to 10% MeOH in DCM, 1% TEA) to yield pure 2'-deoxy-5-carboxymethylcytidine (270 mg, 0.946 mmol, 97%). ^{1}H NMR (400 MHz, D_2O): δ 8.85 (s, 1H), 6.03 (dd, *J* = 6.6, 5.0 Hz, 1H), 4.33 (q, *J* = 5.7 Hz, 1H), 3.99 (q, *J* = 4.1 Hz, 1H), 3.83 (dd, *J* = 12.6, 3.3 Hz, 1H), 3.75 (s, 3H), 3.70 (dd, *J* = 12.6, 4.1 Hz, 1H), 2.50 – 2.39 (m, 1H), 2.22 (ddd, *J* = 13.9, 6.5, 5.1 Hz, 1H); ^{13}C NMR (101 MHz, D_2O) δ 165.78, 163.29, 155.55, 148.69, 96.40, 86.95, 86.76, 69.18, 60.11, 52.42, 40.13; HRMS: Neutral mass for $[C_{11}H_{15}N_3O_6]$ calculated 285.0961, found 285.0969.

Full NMR spectra are provided in Section 3E.

Catalyst preparation

Anion exchange was performed to produce $Ir(dF(CF_3)ppy)_2(dtbbpy)]Cl$: $Ir(dF(CF_3)ppy)_2(dtbbpy)]PF_6$ was dissolved in H_2O/MeCN (30 mL, 1:1). Dowex resin (1X8 chloride form, 50-100 mesh) was washed on a vacuum filter with aqueous NaCl (0.5 M, 3 x 50 mL) and water (50 mL). The dissolved catalyst was filtered five times over the column, which was then washed with water (50 mL). The combined filtrates were lyophilised to obtain the chloride catalyst form as a yellow powder (>95% recovery).

2B – Photoreaction procedures

Supplementary method A: Nucleoside photoreactions

For a representative small-scale screening reaction, the following components were added to a standard 2 mL LC-MS vial: 50 µL buffer (sodium acetate, pH 4.5, 1.0 M), thiol (e.g 2-mercaptoethanol, 35.2 µL, 0.5 mmol), nucleoside stock solution (e.g d5caC, 50 µL, 100 mM in water, 5.0 µmol), **Ir[CF_3]Cl** stock solution (100 µL, 5 mM e.g in MeCN, 0.5 µmol), and the volume made up to 500 µL with water. A stir bar was added and the vial was capped and vortexed briefly to mix. Reactions were incubated with stirring in a PhotoRedOx Box (Hepatochem), with continual illumination by 450 nm/30 W LEDs, under an air atmosphere and at room temperature. Reaction samples taken at desired timepoints were diluted 10x in water and frozen in the interim or analysed immediately by tandem LC-MS (0.5 to 25% MeCN in H_2O, 5 mM NH_4HCO_3).

Supplementary method B: Oligonucleotide photoreactions

For a representative oligonucleotide or DNA test, the following components were mixed in a colourless 0.2 mL PCR tube: 5 µL buffer (sodium acetate, pH 4.5, 1.0 M), fresh 2-mercaptoethanol (1.76 µL), oligonucleotide stock solution (5 µL, 40 ng/µL in water), **Ir[CF_3]Cl** stock solution (10 µL, 0.5 mM in MeCN), and the volume made up to 50 µL with water. Reaction mixtures were vortexed briefly, centrifuged and incubated under ambient atmosphere at room temperature in a PhotoRedOx Box (Hepatochem), with continual illumination by 450 nm, 30 W EvoluChem LEDs. Reactions on DNA 10mers were purified after the desired length of time using mini Quick Spin Oligo Columns (Roche), and diluted 2x with water before LC-MS analysis (5 to 40% MeOH in H_2O, 10 mM TEA & 100 mM HFIP). Reactions on longer oligonucleotides were purified using Oligo Clean & Concentrate spin columns (Zymo).

2C – Next-Generation DNA sequencing procedures

Supplementary method C: Synthetic oligonucleotides

The following 74mer model oligonucleotides (sequences adapted from Taqα1 assay templates[3]) were used as model substrates for single-stranded DNA:

5'-GCTGGGGAACTACAGGCTGACAGTCCGGGCGGTAAATGCG[X]CGAACCCGACGGTACAGTTTGAGTTCTGGTTCT-3'

74mer_caC: X = 5caC (test oligo – modified at base 41)

74mer_C: X = C (control)

1. For each photoreaction, the required components were mixed in a standard 0.5 mL Eppendorf tube. A general example:

Reagent	Volume (µL)	Final amount
Sodium acetate buffer (pH 4.5, 1.0 M)	5	0.1 M
Thiol	0 to 5	0.0 to 1.0 M
Oligonucleotide (40 ng/µL)	5	200 ng
Catalyst stock (0.5 mM in MeCN)	10	0.1 mM catalyst, 20% v/v MeCN
Water	To 50	

Each reaction was vortexed and centrifuged briefly, before incubation under air atmosphere at room temperature in a PhotoRedOx Box (Hepatochem), with continual illumination by 450 nm/30 W LEDs for up to 20 minutes. Reactions were purified using Oligo Clean & Concentrate kits (Zymo) eluting with water.

2. Samples were amplified by PCR using KAPA HiFi Uracil+ polymerase, which reads DHU as T.[3] The following extension primers were used to incorporate standard Read 1/Read 2 sequences:

PCR1_overhang_fwd: 5' - ACACTCTTTCCCTACACGACGCTCTTCCGATCTGCTGGGGAACTACAGG - 3'

PCR1_overhang_rev: 5' - GTGACTGGAGTTCAGACGTGTGCTCTTCCGATCTAGAACCAGAACTCAAACTGTA - 3'

Cycling conditions:

Temperature (˚C)	Time	Cycles
95	3 mins	1
98	20 secs	5
65	15 secs	
72	15 secs	
72	1 min	1

Reaction products were purified using Oligo Clean & Concentrate kits, quantified by Qubit HS dsDNA assay and diluted to 1 ng/µL.

3. Samples were amplified and indexed for sequencing using the universal and index primers from NEBNext Multiplex Oligos for Illumina and Q5 Ultra II polymerase, according to manufacturer's protocols. Purification and quantification was carried out as described in step 2.

4. Indexed samples were normalised, pooled and analysed by single-end sequencing with Phix Control v3 spike-in (50%).

5. Fastq files were quality checked (*fastQC*, v0.11.9) and clipped with bash *awk* (awk 4.0.2, zcat `$file| paste - - - - | awk -v FS="\t" -v OFS="\n" '$2 ~ "^GCTGGGGAACTACA.*TTGAGTTCTGGTTCT$" {print $1, $2, $3, $4}'`). Clipped reads were aligned to the 74 bp-long reference sequence (74mer_caC) using *bowtie* (1.3.0) with default options, and alignments were saved as bam files using *samtools view* (1.11). Mutation calls were performed using a custom bash script that uses *bsftools mpileup* (*bcftools* 1.9,Using *htslib* 1.9). The output of *mpileup* was parsed in order to extract the number of each base type (A, G, T, G) present across all reads at each of the 74 reference positions. The fraction of each base was obtained as the total number of that base divided by the total number of reads covering the locus. To obtain the average and median frequency by base type, the frequency of all bases of the same type (i.e. C bases, A bases, …) was combined with the appropriate formula. Position 41 (containing the modified 5caC base) was treated separately from all other C bases in the reference oligo: it was excluded in the computation of the statistics relative to C bases. Custom R scripts employing statistical base functions, dplyR and ggplots2 were designed and used to generate bar plots of individual base frequencies and plots and statistics of the combined averages of frequencies. The background mutation rate of untreated samples (a product of synthetic impurities in oligo and errors in PCR/sequencing) was subtracted from test samples to obtain the off-target conversions induced by chemical treatments.

Supplementary method D: λ-phage genomic DNA

1. Unmethylated λ-phage DNA was artificially methylated at CpG dinucleotide sites using commercial M.SssI (Zymo) according to manufacturer's protocols. The product was purified using Ampure XP beads (1.6X) and methylation confirmed *via* HpaII protection assay.

2. 5mC oxidation was carried out using TET2 enzyme obtained from an NEBNext Enzymatic Methyl-seq Conversion Module (NEB) (n.b: NgTet1 supplied by Active Motif was also tested but DNA recovery was poor). Reactions were prepared and incubated as below*:

Reagent	Volume (µL)	Final amount
Methylated λ-phage DNA in nuclease-free water	30	200 ng
TET2 reaction buffer (10X)	10	1X
DTT	1	2% v/v
TET2 enzyme	4	(-)
Fe^{II} solution (400 µM)	5	40 µM
Total	50 µL	

**The* Oxidation Supplement *and* Oxidation Enhancer *provided as part of the Conversion Module were omitted from the reactions.*

Oxidised DNA was purified using Ampure XP beads (1.8X), sheared *via* ultrasonication and size-selected to 200bp fragments.

3. KAPA Hyper Prep Kit reagents were used according to manufacturer's protocols for A-tailing, end-repair and adapter ligation, using full-length xGen UDI-UMI methylated adapters. Ampure XP beads (1.4X) were used for post-ligation clean-up with elution using nuclease-free water.

4. Chemical treatments were carried out on the prepared DNA: Prior to photochemical treatment, library molecules were converted to single-stranded DNA: 100 ng portions of each indexed library were diluted with water to 30 µL, heated to 95˚C for 4 minutes, centrifuged briefly and immediately placed in a tube rack pre-cooled to -80˚C. Reagents for parallel photochemical reactions (as described in Supplementary Method C) were mixed and overlaid on the frozen DNA samples, pipetted while melting and immediately illuminated. In certain

specified cases, a further hydrolysis step was applied: 50 µL of acetate buffer (pH 4.5, 1 M) was added to the 50 µL reaction sample which was incubated in the dark at 37˚C for the desired number of hours.

5. Samples were purified using Oligo Clean & Concentrate columns and amplified using KAPA HiFi Uracil+ polymerase with xGen Library Amplification Primer Mix. PCR products were purified with DNA Clean & Concentrate spin columns (Zymo).

Cycling conditions:

Temperature (˚C)	Time	Cycles
98	1 mins	1
98	15 secs	10
60	30 secs	
72	30 secs	
72	30 secs	1

6. Library size distributions and concentrations were measured by TapeStation, normalised, and samples were pooled for sequencing. The combined library was re-purified with Ampure XP beads (0.8X) to remove adapter-dimers before analysis by paired-end sequencing with PhiX Control v3 spike-in (15%).

7. BCL files were demultiplexed with *bcl2fastq* (2.20.0.422) to account for and identify molecular barcodes in addition to Illumina adaptors. Base quality was assessed with *fastQC* and reads were trimmed to remove Illumina adaptors using *trimgalore* (0.6.6). The resulting reads were aligned using *astair align* (3.3.2) with default options in the case of photochemically-treated libraries (to study 5caC to T) or with --method CtoT in case of bisulfite treated samples. Duplicate removal was performed based on UMI sequences using *UMI-tools* (1.1.2). A custom bash script calling bcftools mpileup was used to count the number of observed bases of each type across the aligned deduplicated reads (forward and reverse) at each individual genomic locus of the λ genome. For libraries where 5caC to T mutation was expected, mutation rates for C bases (in both CpG or not CpG) contexts were obtained by assessing the number of T over the total number of C and T across all the aligned reads. If the reference genome possessed a C on the forward strand, the mutation rate was obtained as the total number of Ts on reverse strand versus the sum of Cs and Ts on reverse; vice versa, if the C was present on the reverse strand of the reference genome, the total number of forward occurrences of Ts and Cs were used to determine the mutation rate. In the case of bisulfite-treated libraries, if the reference genome had a C base on forward strand, the mutation rate was obtained as total number of Cs divided the sum of Cs and Ts from the reverse aligned reads at that given locus; if the C base was on the reverse strand, the forward aligned reads were used for this assessment. Mutations rates for all other bases were obtained as the number of unintended base occurrences (i.e any non-consensus base read at a given genomic position) over the total number of aligned reads. The resulting data were parsed with a custom R script that summarized genome-wide mutations rates by producing the mean and standard error of the mean for each base-context (CpG, non-CpG, A, T, G).

3 – Experimental data

3A – Exploration of d5caC nucleoside reactivity

Key LC-MS chromatograms

UV chromatograms (260 nm absorbance) of crude photoreaction mixtures. Consumption of d5caC was estimated from changes in the integrated nucleoside absorbance relative to internal dA standard. Experiments were carried out once.

Figure S1

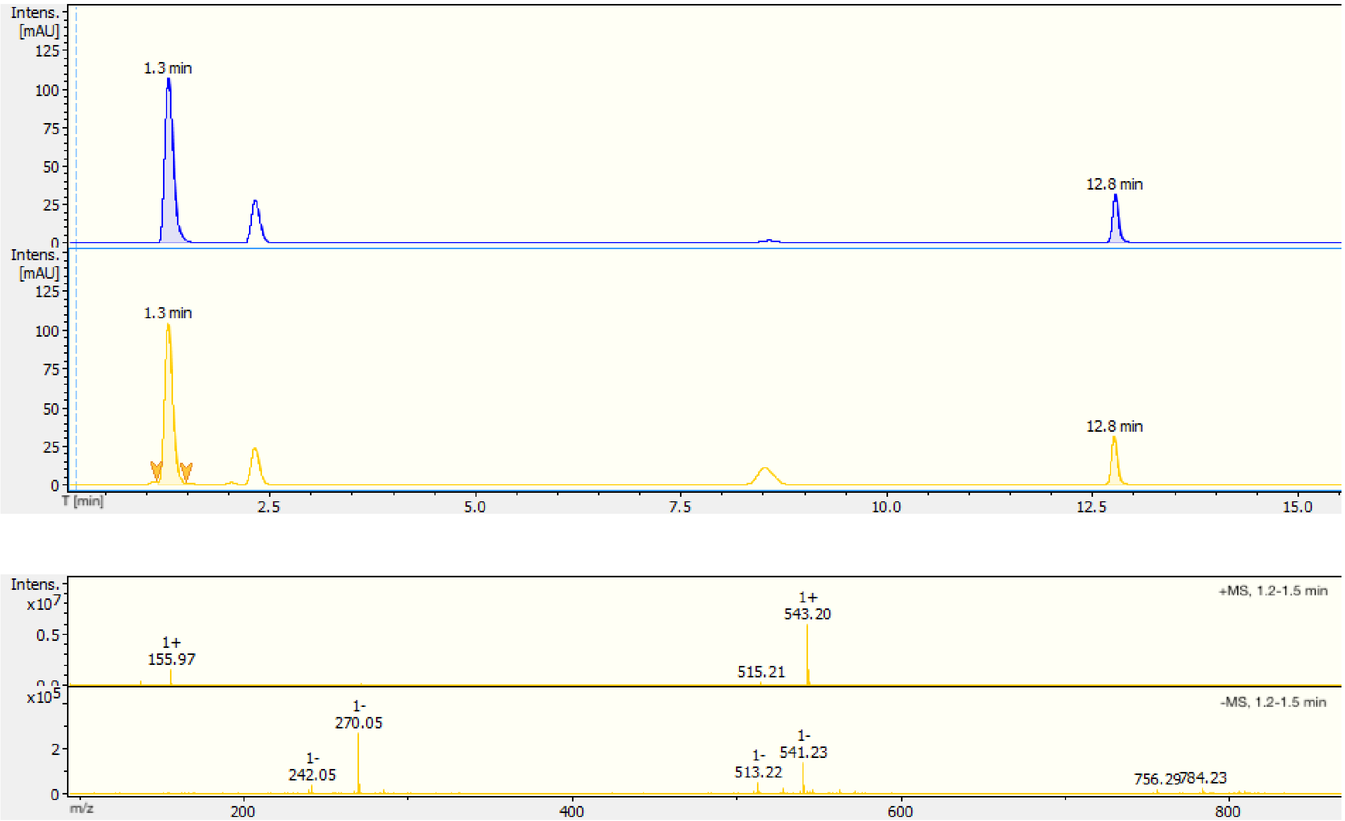

UV chromatograms of d5caC reaction prepared according to Supplementary Method A, using 10 mM nucleoside, 250 mM 2-mercaptoethanol, 1.0 mM **Ir[CF$_3$]Cl**, 100 mM NaOAc (pH 4.5) in H_2O. Reaction sampled at T = 0 h (upper trace: d5caC/dA = 4.93) and T = 3 h (middle trace: d5caC/dA = 4.56). Peaks correspond to d5caC (1.3 mins), dA (12.8 mins), and 2-mercaptoethanol thiol/by-products (2.4 & 8.5 mins). d5caC absorbance was reduced by 7.4% after 3 hours' incubation. Extracted mass spectra at d5caC peak (bottom spectra) showing the nucleoside ($[M-H]^-$ = 270.05), deglycosylated nucleobase ($[M+H]^+$ = 155.97) and nucleoside dimer ($[M+H]^+$ = 543.20).

Figure S2

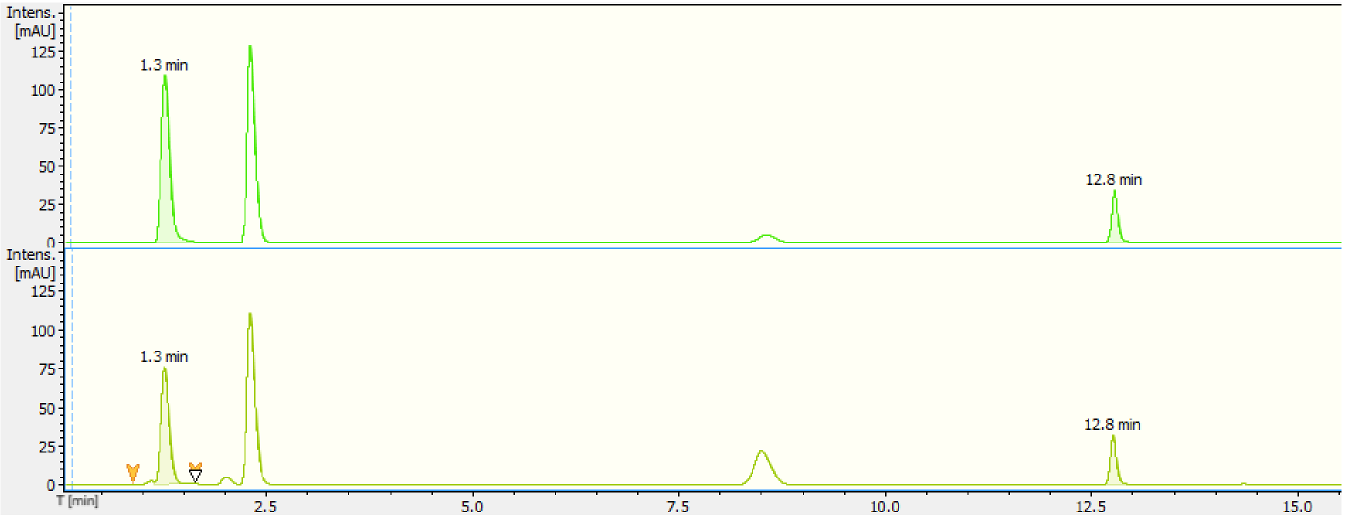

UV chromatograms of d5caC reaction prepared according to Supplementary Method A, using 10 mM nucleoside, 1.0 M 2-mercaptoethanol, 1.0 mM **Ir[CF$_3$]Cl**, 100 mM NaOAc (pH 4.5) in H_2O. Reaction sampled at T = 0 h (upper trace: d5caC/dA = 4.55) and T = 3 h (lower trace: d5caC/dA = 3.13). Peaks correspond to d5caC (1.3 mins), dA (12.8 mins), and 2-mercaptoethanol thiol/by-products (2.4 & 8.5 mins). d5caC absorbance was reduced by 31.2% after 3 hours' incubation.

Figure S3

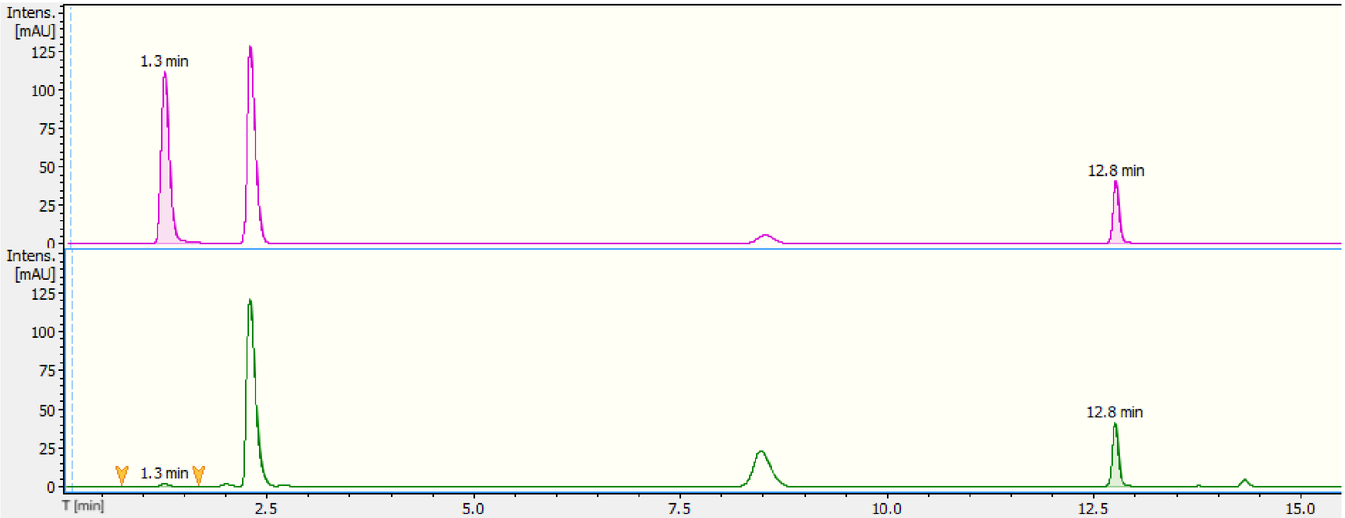

UV chromatograms of d5caC reaction prepared according to Supplementary Method A, using 10 mM nucleoside, 1.0 M 2-mercaptoethanol, 1.0 mM **Ir[CF$_3$]Cl**, 100 mM NaOAc (pH 4.5) in H_2O including 20% v/v acetonitrile. Reaction sampled at T = 0 h (upper trace: d5caC/dA = 3.85) and T = 3 h (lower trace: d5caC/dA = 0.06). Peaks correspond to d5caC (1.3 mins), dA (12.8 mins), and 2-mercaptoethanol thiol/by-products (2.4 & 8.5 mins). d5caC absorbance was reduced by 98.4% after 3 hours' incubation.

Figure S4

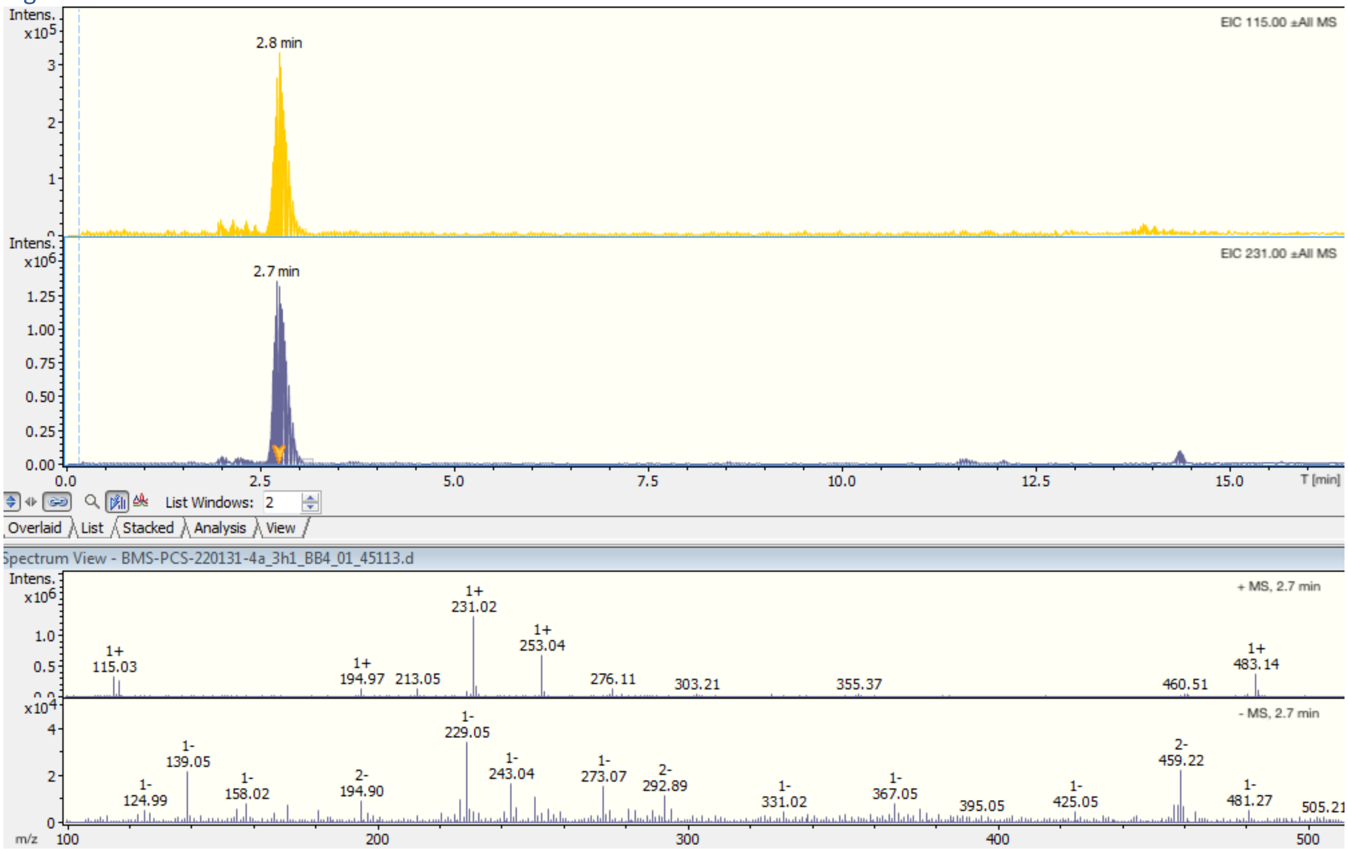

Extracted mass chromatograms for 115 m/z and 231 m/z from the 3-hour sample of d5caC reaction described in Figure S3. A mass fingerprint indicative of the presence of 2'-deoxy-5,6-dihydrouridine (DHU) was detected at 2.7 mins: DHU nucleoside (detected $[M+H]^+ = 231.02$), nucleobase (detected $[M+H]^+ = 115.03$), nucleoside dimer (detected $[M-H]^- = 459.22$):

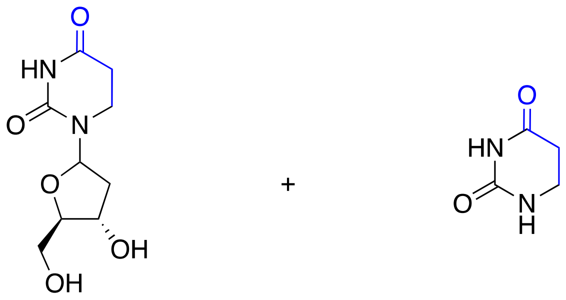

$C_9H_{14}N_2O_5$ $C_4H_6N_2O_2$

Predicted m/z: 231.22 Predicted m/z: 115.10

Isolation & characterisation of DHU product

Eight 500-µL reactions were prepared as described above, illuminated in parallel overnight, combined, and evaporated to dryness *in vacuo*. The residue was redissolved in water (1.5 mL) and purified by reversed-phase HPLC (0 to 10% MeCN in H_2O, 0.1% TFA). After lyophilisation, the product was obtained as a white solid (2.1 mg, 9.13 µmol). The spectral data were found to match those obtained from a commercial reference, confirming the product to be 2'-deoxy-5,6-dihydrouridine: ^{1}H NMR (500 MHz, D_2O): δ 6.15 (ddd, *J* = 8.1, 5.0, 1.8 Hz, 1H), 4.26 (ddt, *J* = 7.5, 5.5, 2.7 Hz, 1H), 3.80 (dtd, *J* = 5.7, 3.9, 1.8 Hz, 1H), 3.70 – 3.62 (m, 1H), 3.60 – 3.53 (m, 1H), 3.48 – 3.36 (m, 2H), 2.64 (dddd, *J* = 8.6, 6.8, 5.1, 1.9 Hz, 2H), 2.25 – 2.15 (m, 1H), 2.02 (dddd, *J* = 14.2, 8.2, 3.8, 1.9 Hz, 1H); 13C NMR (500 MHz, D2O): 173.85, 154.18, 85.10, 83.97, 70.77, 61.50, 35.65, 35.13, 30.00; HRMS $[M-H]^-$ for $[C_9H_{13}N_2O_5]^-$ calculated 229.0824, found 229.0834. Full spectra are provided in Section 3E.

Further LC-MS chromatograms: Reaction development

Table S1: UV data of d5caC reactions using different organic co-solvents

Co-solvent	d5caC/dA (t = 0 h)	d5caC/dA (t = 5 h)	5caC consumption (%)
THF	1.086	0.202	81.4
DMSO	1.175	0.029	97.6
1,4-dioxane	1.167	0.043	96.3
1,2-ethanediol	0.982	0.012	98.8

Three-hour reactions without co-solvent and with MeCN are demonstrated in figures S2 and S3.

Nucleoside tests

UV chromatograms in relation to the experiments described in Table 3. Consumption of nucleosides was estimated from changes in the integrated UV absorbance relative to internal dA standard. Experiments were carried out twice.

Figure S5

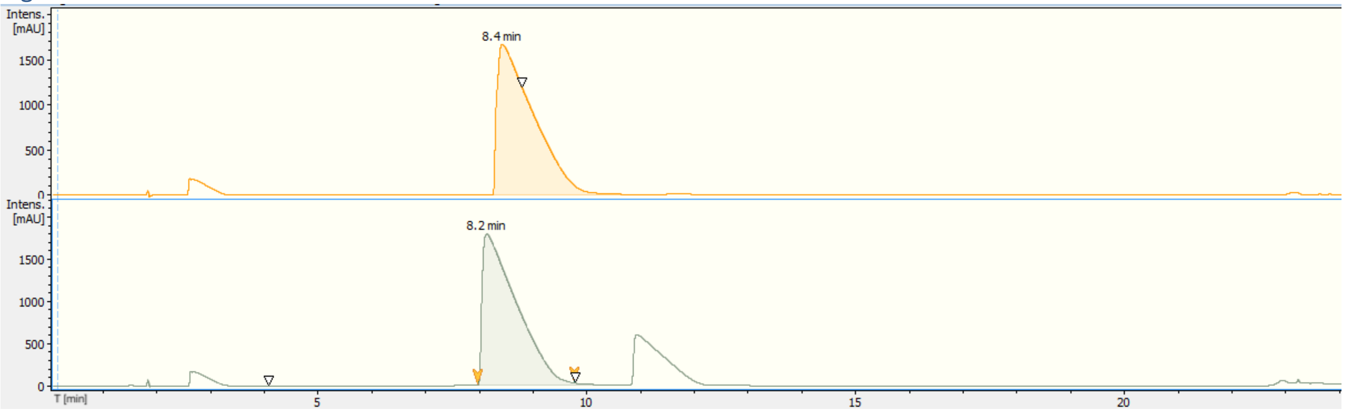

UV chromatograms of dA reaction prepared according to Supplementary Method A, sampled at T = 0 h (upper) and T = 3 h (lower). Peaks correspond to dA (8.2-8.4 mins) and 2-mercaptoethanol thiol/by-products (2.8 + 11.0 mins).

Figure S6

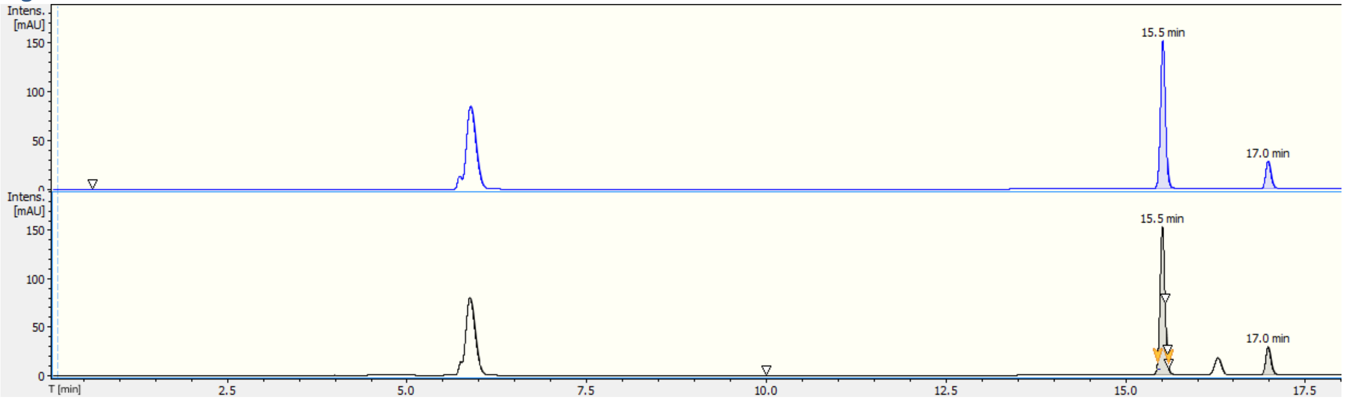

UV chromatograms of dT reaction prepared according to Supplementary Method A, sampled at T = 0 h (upper) and T = 3 h (lower). Peaks correspond to dT (15.5 mins), 2-mercaptoethanol thiol/by-products (5.8 + 16.3 mins) and dA (17.0 mins).

Figure S7

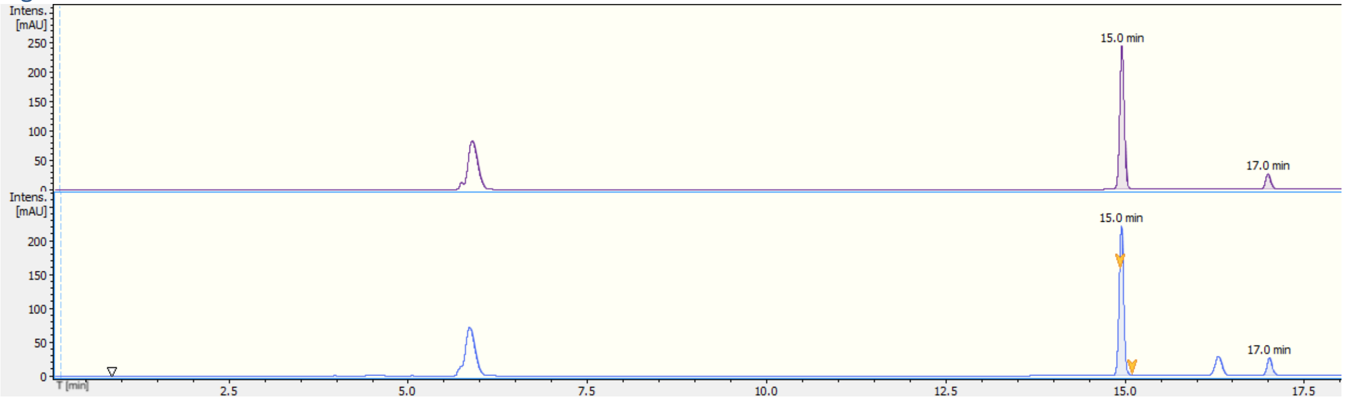

UV chromatograms of dG reaction prepared according to Supplementary Method A, sampled at T = 0 h (upper) and T = 3 h (lower). Peaks correspond to dG (15.0 mins), 2-mercaptoethanol thiol/by-products (5.8 + 16.3 mins) and dA (17.0 mins).

Figure S8

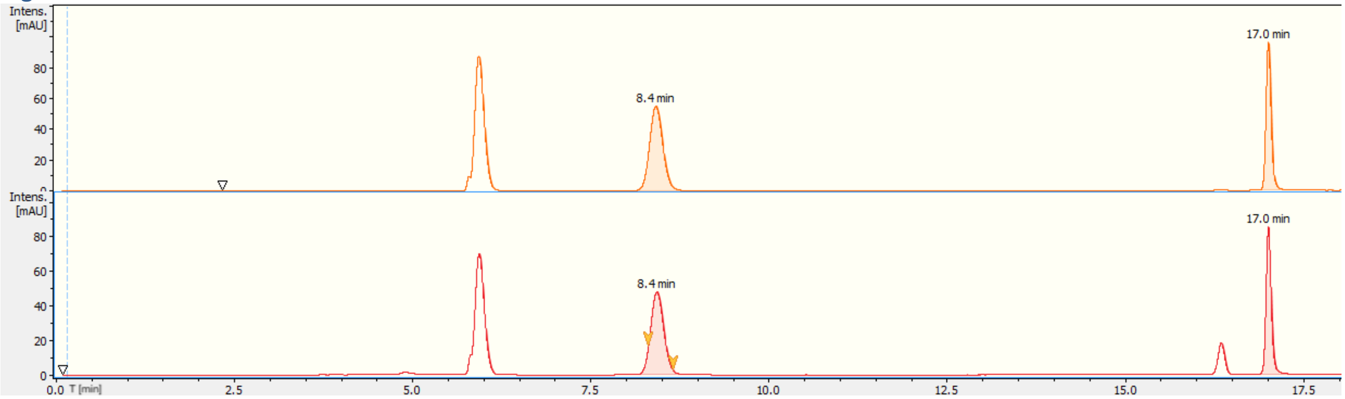

UV chromatograms of dC reaction prepared according to Supplementary Method A, sampled at T = 0 h (upper) and T = 3 h (lower). Peaks correspond to dC (8.4 mins), 2-mercaptoethanol thiol/by-products (5.9 + 16.4 mins) and dA (17.0 mins).

Figure S9

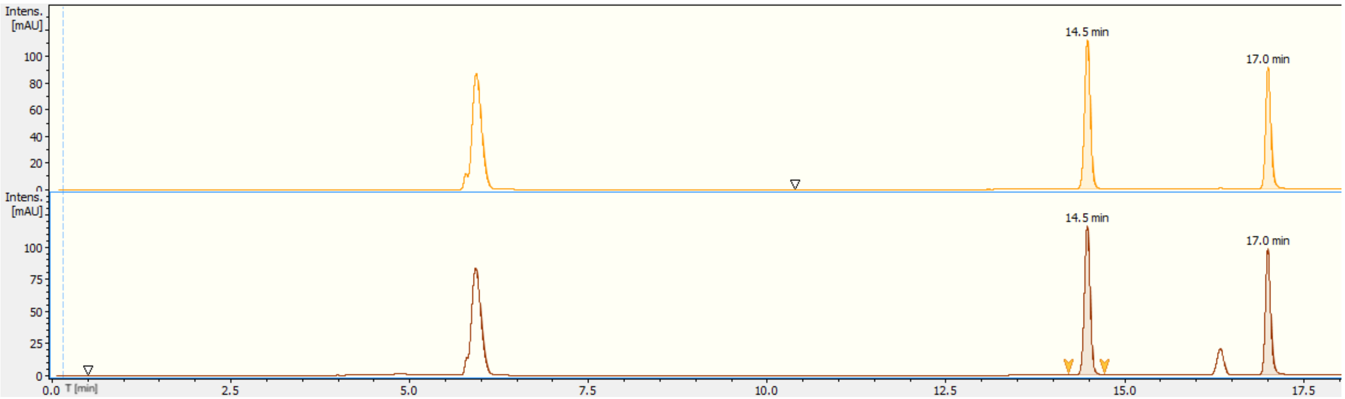

UV chromatograms of d5mC reaction prepared according to Supplementary Method A, sampled at T = 0 h (upper) and T = 3 h (lower). Peaks correspond to d5mC (14.5 mins), 2-mercaptoethanol thiol/by-products (5.9 + 16.4 mins) and dA (17.0 mins).

Figure S10

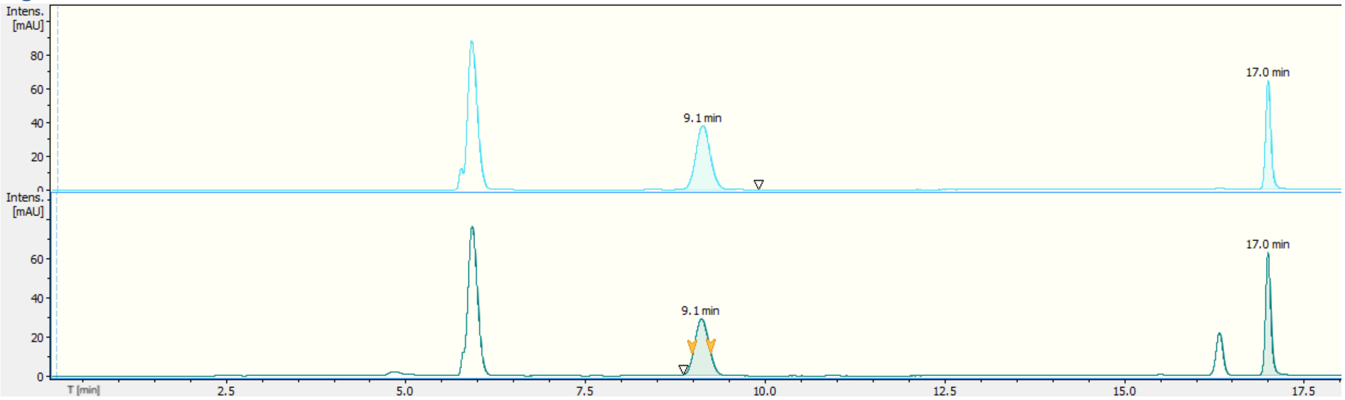

UV chromatograms of d5hmC reaction prepared according to Supplementary Method A, sampled at T = 0 h (upper) and T = 3 h (lower). Peaks correspond to d5hmC (9.1 mins), 2-mercaptoethanol thiol/by-products (5.9 + 16.4 mins) and dA (17.0 mins).

Figure S11

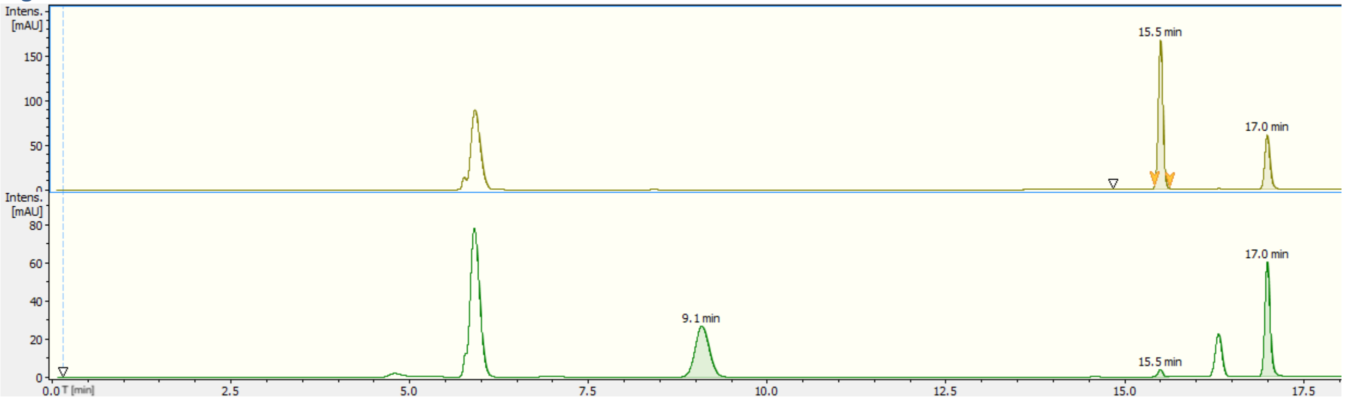

UV chromatograms of d5fC reaction prepared according to Supplementary Method A, sampled at T = 0 h (upper) and T = 3 h (lower). Peaks correspond to d5hmC (9.1 mins, characterized by ESI LC-MS without isolation), d5fC (15.5 mins), 2-mercaptoethanol thiol/by-products (5.9 + 16.4 mins) and dA (17.0 mins).

Figure S12

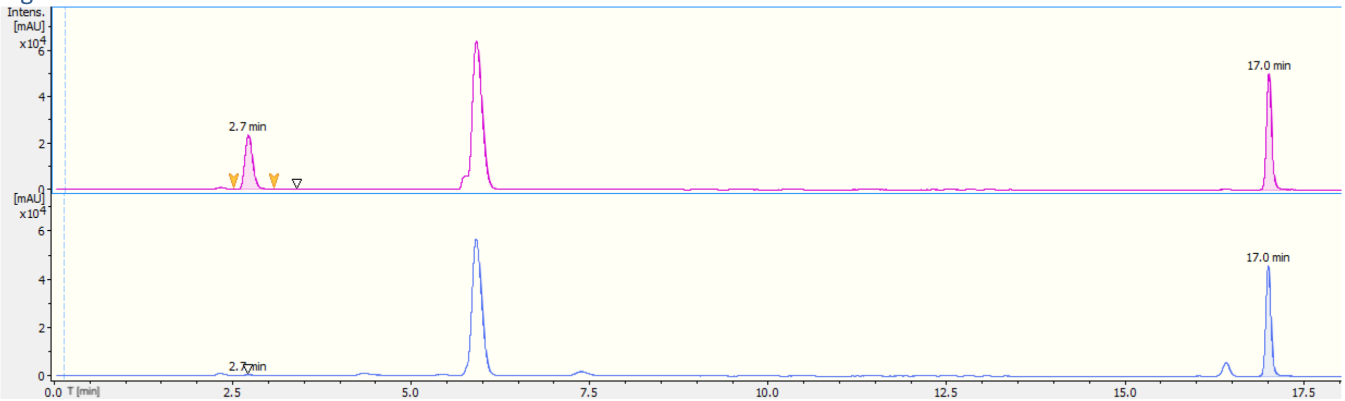

UV chromatograms (200-300 nm) of d5caC reaction prepared according to Supplementary Method A, sampled at T = 0 h (upper) and T = 3 h (lower). Peaks correspond to d5caC (2.7 mins), 2-mercaptoethanol thiol/by-products (5.9 + 16.4 mins) and dA (17.0 mins).

Table S2: UV data of reactions of different nucleosides

Nucleoside	dA integral (t = 0 h)	dA integral (t = 3 h)	Concentration change (%)	Notes
dA	79195	80730	1.9 ± 1.1[a]	*Determined from absolute integrals of dA*
	dN/dA (t = 0 h)	dN/dA (t = 3 h)	Consumption (%)	*Determined from ratio of integrals of dN to dA*
dT	5.023	4.993	-0.6 ± 0.1	*Thymidine contains O instead of N at C4 and cannot undergo deamination*
dG	3.223	3.062	-8.2 ± 0.1	*Minor oxidation to 8-oxo-G*
dC	1.446	1.413	-2.3 ± 0.2	
d5mC	1.333	1.302	-2.3 ± 0.0	
d5hmC	1.598	1.257	-21.3 ± 0.8	*No deamination/reduction products detected*
d5fC	2.454	0.035	-98.6 ± 1.3	*Reaction product is species eluting at 9.1 mins with m/z 257.8 & 515.1 (d5hmC). No deamination products detected*
d5caC	0.684	0.007	-98.9 ± 0.9	

Nucleoside consumption is reported as the average of two replicates ± standard deviation. [a]dA was deemed practically inert (mean of n = 4; change is within bounds of error of analysis *via* LCMS integration) on this timescale, and thereafter used as an internal reference in experiments with other nucleosides.

3B – Mechanistic study

Substrate analogue

Figure S13

A

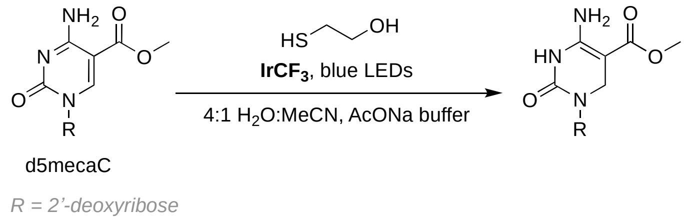

B

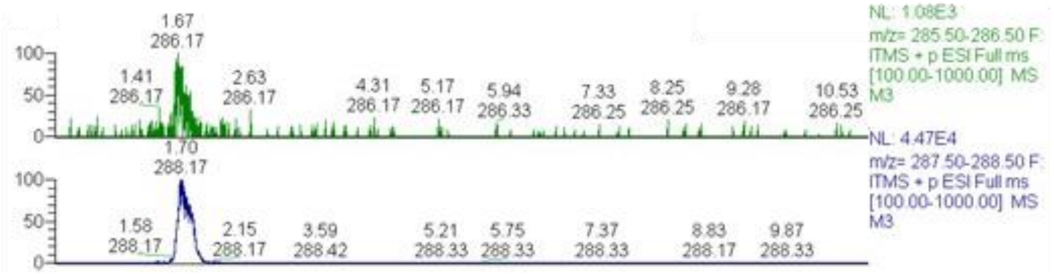

C

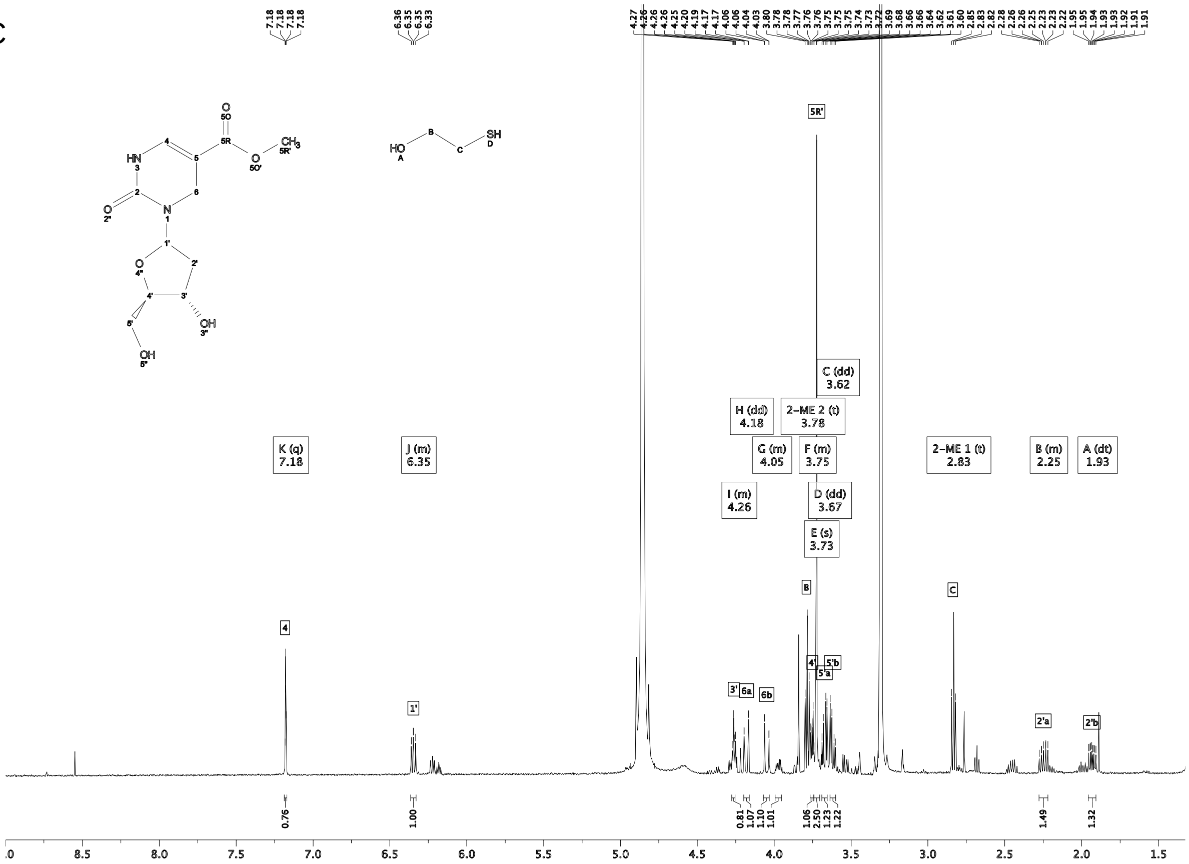

C5-C6 reduction without decarboxylation was observed on the d5mecaC nucleoside by LC-MS analysis (Fig. S13A & S13B), and a second product corresponding to double-reduction and loss of the C4 amine was identified by LC-MS and NMR (Fig. S13C).

Stern-Volmer analysis

Solutions of photocatalyst and quenchers were prepared in the reaction solvent (20% MeCN in H_2O, with 100 mM pH 4.5 sodium acetate buffer). The concentration of [Ir(dF(CF$_3$)ppy)$_2$(dtbbpy)]Cl was 10 µM. Samples were irradiated at 450 nm and emission measured at 500 nm. Data points represent an average of two measurements.

Figure S14

Stern-Volmer plot of [Ir(dF(CF$_3$)ppy)$_2$(dtbbpy)]Cl (10 µM) fluorescence with increasing concentrations of 2'-deoxy-5-methylcarboxycytidine.

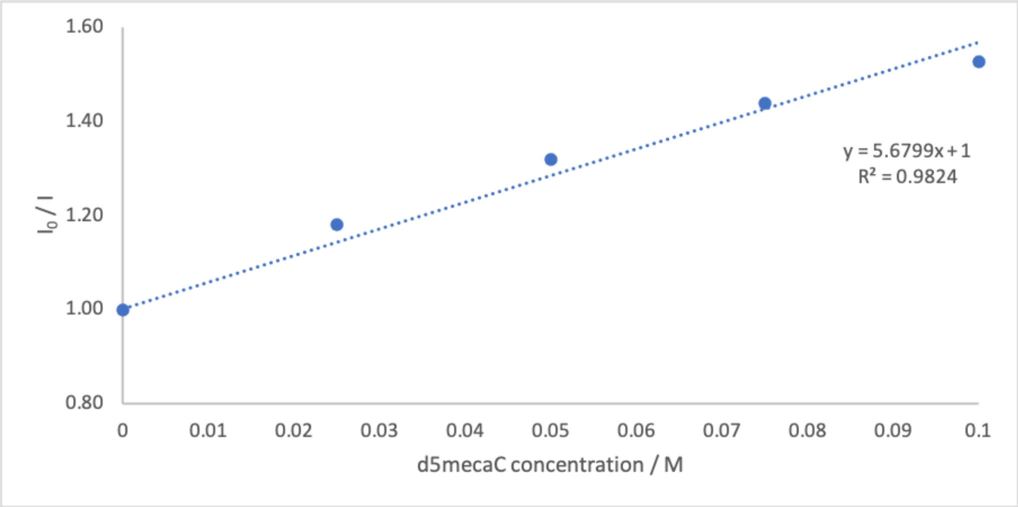

Figure S15

Stern-Volmer plot of [Ir(dF(CF$_3$)ppy)$_2$(dtbbpy)]Cl (10 µM) fluorescence with increasing concentrations of 2'-deoxy-5-carboxycytidine.

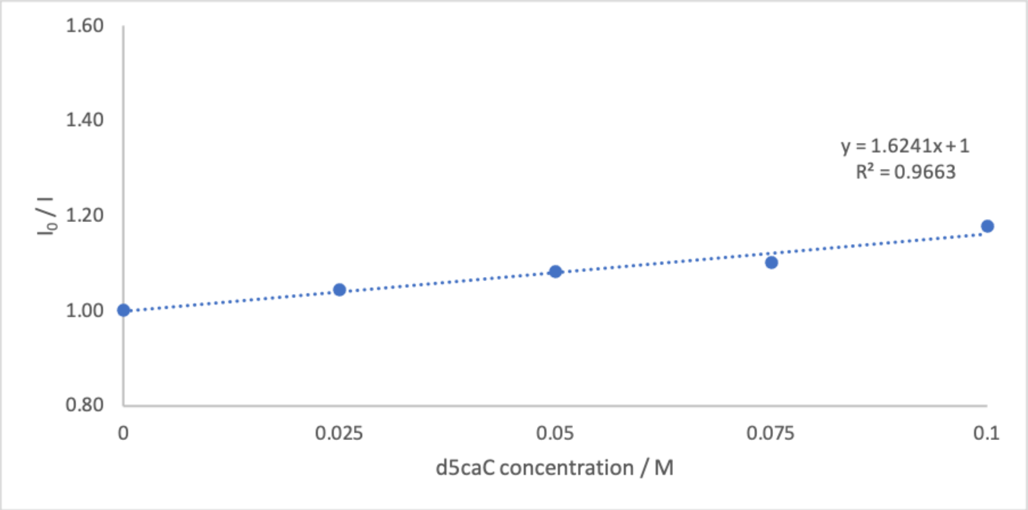

Since the chemical functionality of canonical pyrimidine dC makes no quenching interactions with the excited photocatalyst, the quenching observed selectively with d5caC and d5mecaC is due to an interaction with the carbonyl substituents and/or the electron-deficient nucleobase ring.

Figure S16

Stern-Volmer plot of [Ir(dF(CF_3)ppy)$_2$(dtbbpy)]Cl (10 μM) fluorescence with increasing concentrations of 2'-deoxycytidine.

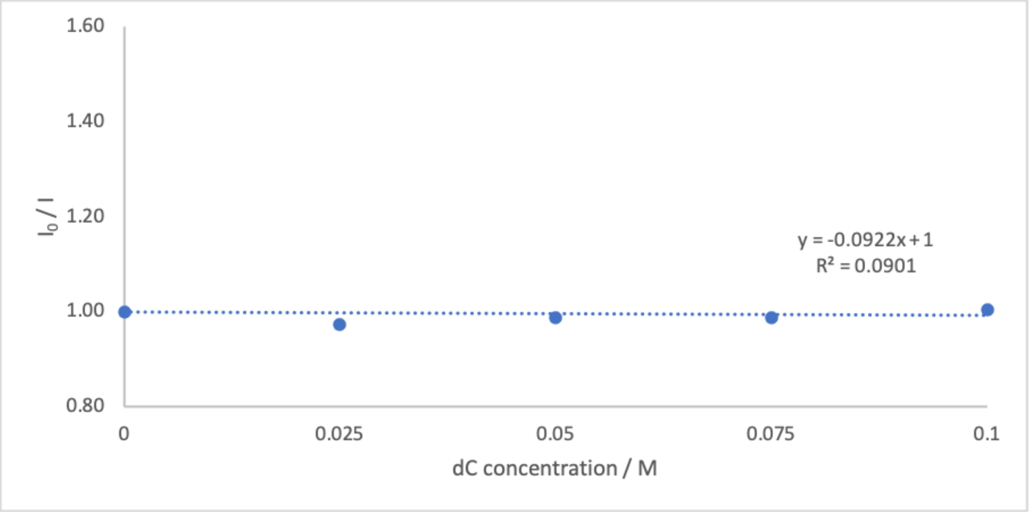

Computational study

All DFT calculations were carried out in Orca 5.0.,[4] using Def2-TZVP[5] basis set with def2/J[6] auxiliary basis, with M06-2x (with increased integration grid, defgrid3), PBE, TPSSh functionals as found in GMTKN30 database.[7] Redox potential estimation followed previously described calculations based on the Born-Haber cycle[8] and Nernst equation (Figure S17) with geometry optimization and Gibbs free energy calculation of neutral and anionic species for all molecules in gas phase as well as in implicit solvent (CPCM water).[9] Results for M06-2x are presented in Table S3. We compared 2-mercaptoethanol, cysteamine (protonated), and thioacetic acid thiols (X) computationally at the MP2/cc-pVTZ level of theory in implicit water. Bond dissociation energies (BDE) for the S-H bonds were calculated by the difference of Gibbs free energy between X and (X-H)$^{\cdot}$ +H$^{\cdot}$. Electronic densities at S were calculated by Hirshfeld analysis. This set of calculations was intended only to compare thiols at the same level of theory and does not represent a detailed computational analysis. These results are presented in Table S4.

Figure S17

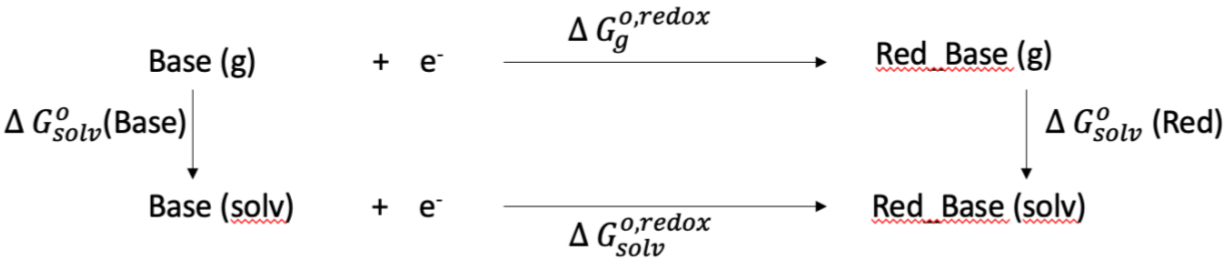

$$E_{Base,red} = \frac{-\Delta G_{solv}^{o,redox}}{nF} - 4.28$$

Cyclic voltammetry

Single-electron reduction potentials were measured using solutions of nucleoside (10 mM) and tetrabutylammonium hexafluorophosphate electrolyte (0.1 M) in dry DMF. Test solutions were sparged with nitrogen for 5 minutes and the reduction potentials were recorded with a glassy carbon working electrode and platinum counter electrode relative to an aqueous silver/silver chloride (Ag/AgCl) reference electrode. A voltage sweep from 0 to -3 V was first performed at 0.1 V/s, before 5 mg ferrocene was added, the solution sparged for a further minute, and a second voltage sweep carried out from 0 to +1 V. Nucleoside reductions were recorded as the half-peak potential, converted relative to the normal hydrogen electrode (NHE) by adding 0.21 V to the measured values, and referenced to the internal ferrocene standard (E = 0.42 V vs NHE).[10]

Table S3: Reduction potentials of nucleosides (V vs NHE)

Nucleoside	Measured potential	Calculated potential	Literature value[11]
dC	-2.25	-2.52	-2.21
d5caC	-1.71	-1.76	n/a
d5mecaC	-1.78	-1.92	n/a
dU	-2.09	-2.26	-2.02
dT	-2.12		-2.12
dA	- 2.45		-2.43
dG	< -2.7 *(reduction not complete within voltage limit)*		< -2.74

Dipole moment and electrophilic susceptibility were calculated and plotted using Gabedit 2.5.0 based on the ORCA extended output file (Figure Comp2).[12]

Figure S18

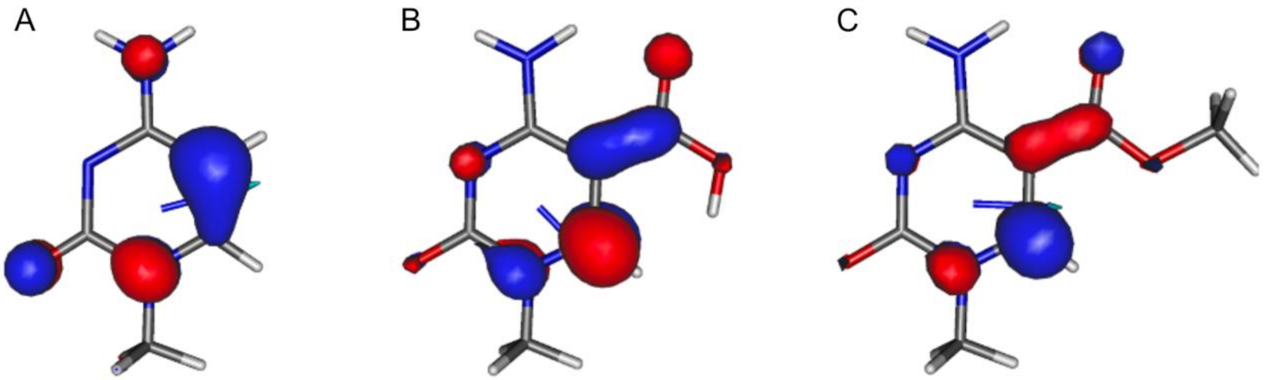

Isosurface plots of the radical anion SOMOs for 1-methylcytosine (A), 1-methyl-5-carboxycytosine (B) and 1-methyl-5-methylcarboxycytosine (C). In the case of (B) and (C), the greatest electron density is localised on the C6 carbon, which is observed to undergo further reaction *via* hydrogen atom transfer.

Figure S19

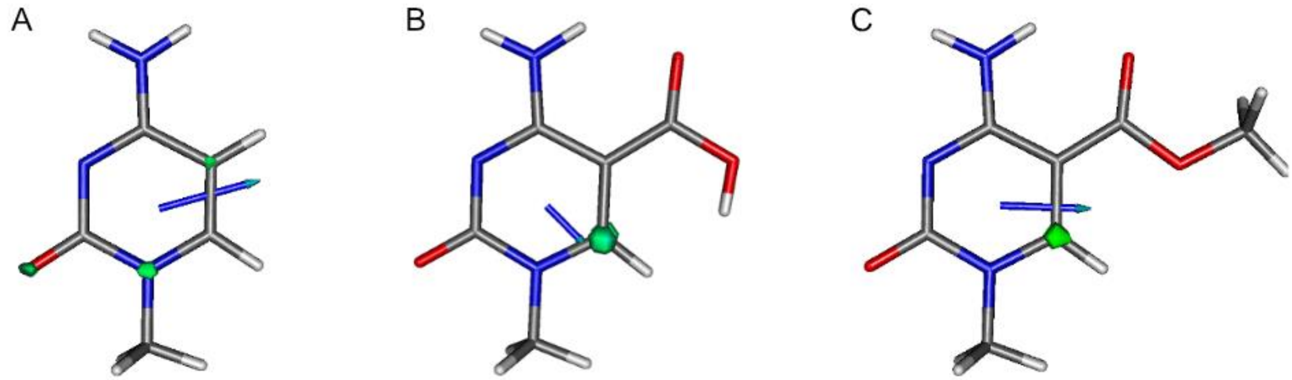

Electrophilic susceptibility plots for 1-methylcytosine (A), 1-methyl-5-carboxycytosine (B) and 1-methyl-5-methylcarboxycytosine (C). Green surfaces map the highest values of the Fukui index (location of susceptibility to further reaction) derived from frontier molecular orbitals.[13]

Thiol rate comparisons, S-H bond dissociation energies and charge calculations

Table S4: BDEs and electrophilicity of thiol functional groups

Thiol	Calculated BDE (kcal mol^{-1})	Hirshfield charge
Cysteamine (protonated)	83.499571	-0.103238
Thioacetic acid	83.615579	-0.059571
2-mercaptoethanol	84.847782	-0.124415

Figure S20

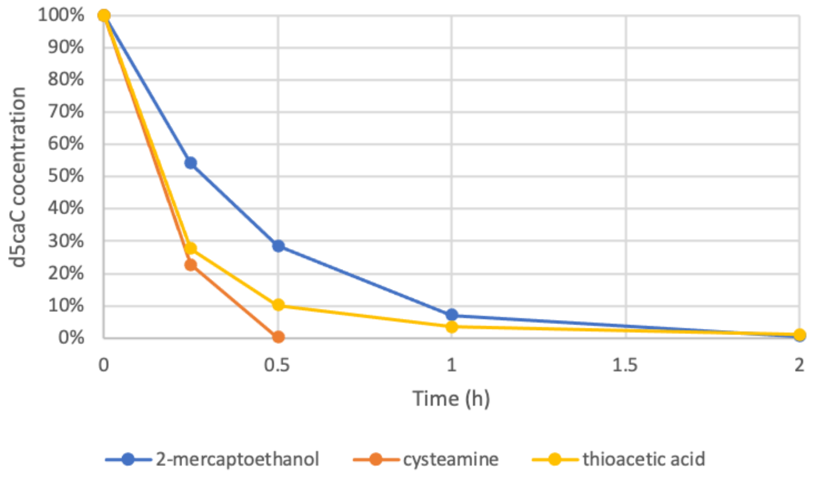

Nucleoside experimental timecourses using different thiols, in reactions prepared according to Supplementary Method A using 10 mM nucleoside, 1.0 M thiol, 1.0 mM **Ir[CF_3]Cl**, 100 mM NaOAc (pH 4.5) in H_2O with 20% v/v acetonitrile.

3C – Reactions on model oligonucleotides

Reactions with model 10mer oligonucleotides were carried out and monitored according to Supplementary Method B (using 0.5 M 2-mercaptoethanol and 0.1 mM **Ir[CF$_3$]Cl** photocatalyst). In each case, quantitative conversion of the starting material was observed. The 10mer model nucleotides consisted of the following sequence *(N = C, G, T, A)*, covering all four "CpN dinucleotide" contexts:

5' – CTTAC[5caC]NTGA – 3'

Selected example:

Figure S21

(5caCpC oligonucleotide, N = C)

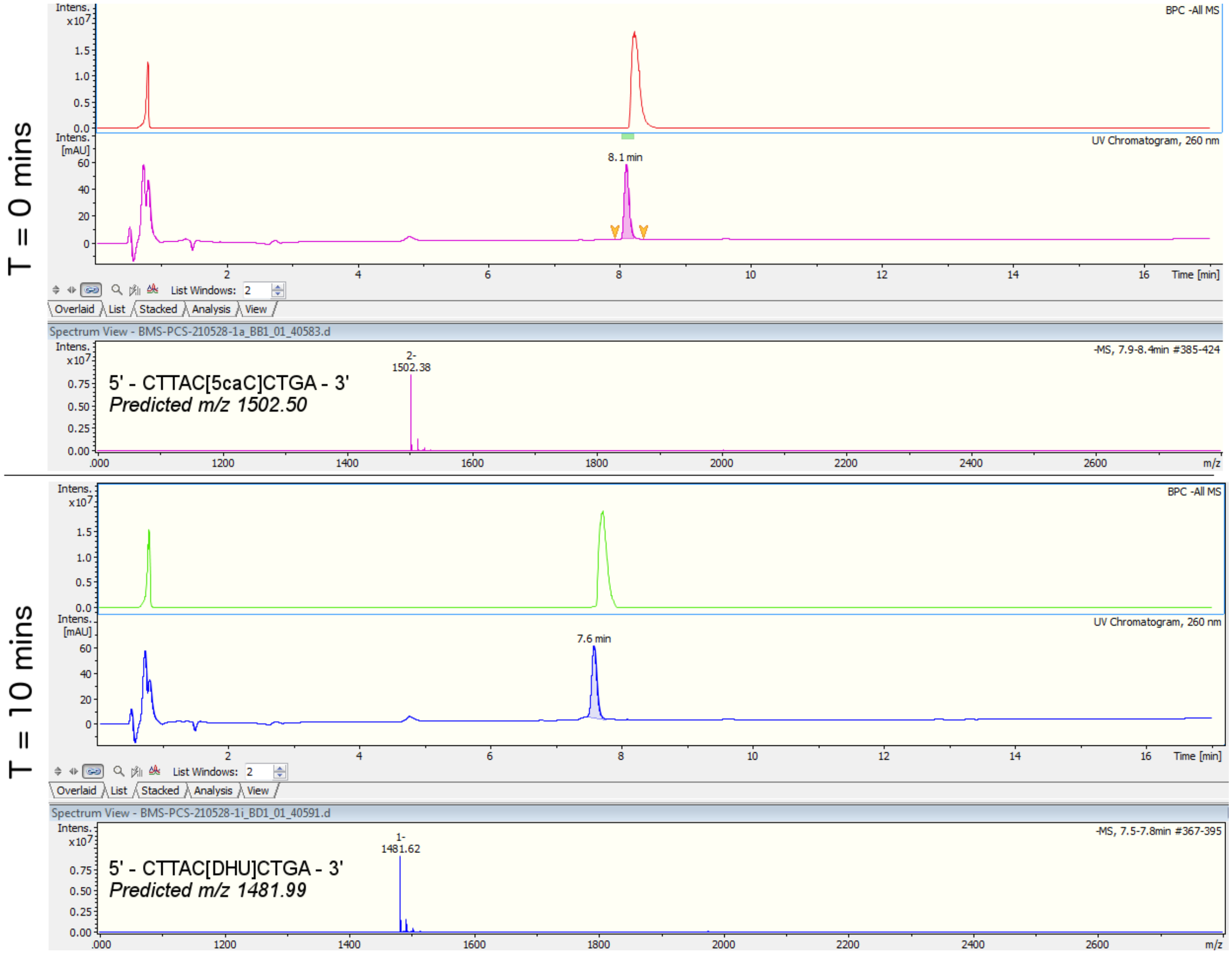

3D – Next-generation sequencing

During PCR, DHU sites are read as 'T' by polymerases due to their hydrogen-bonding pattern. Therefore, sites originally containing 5caC are selectively converted to T upon photochemical treatment followed by amplification and sequencing. In contrast to bisulfite treatment, unmodified cytosine is not converted in the reaction and is detected as C (Table S4), preserving the complexity of the DNA sequence. The photoreaction buffer conditions do not induce significant strand depurination within the short photochemical incubation period; similar conditions have been established by other non-destructive methods such as pyridine borane treatment, which involves incubation at pH 4.3/37˚C for multiple hours.[3]

Synthetic oligonucleotides

Photoreactions were carried out according to Supplementary Method C. Samples were amplified by PCR with a uracil-tolerant polymerase using overhanging primers to incorporate standard adaptor and index sequences, and Illumina sequencing was then performed on the resulting. Sequenced reads of good quality were trimmed to remove Illumina adapters and aligned to the oligonucleotide reference. Mutation calls based on aligned reads were performed at all C bases, as well as at all other base types to assess off-target effects.

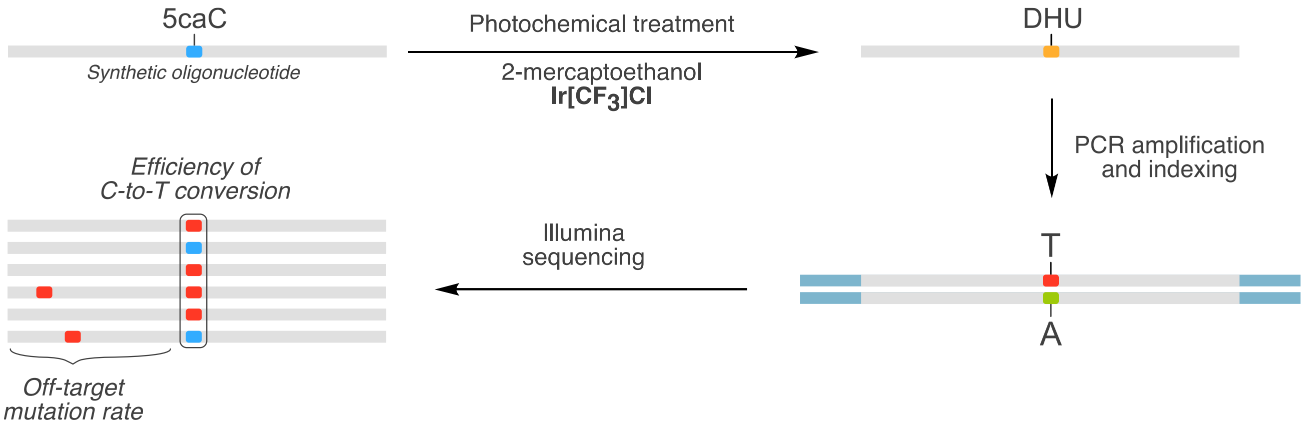

Figure S22

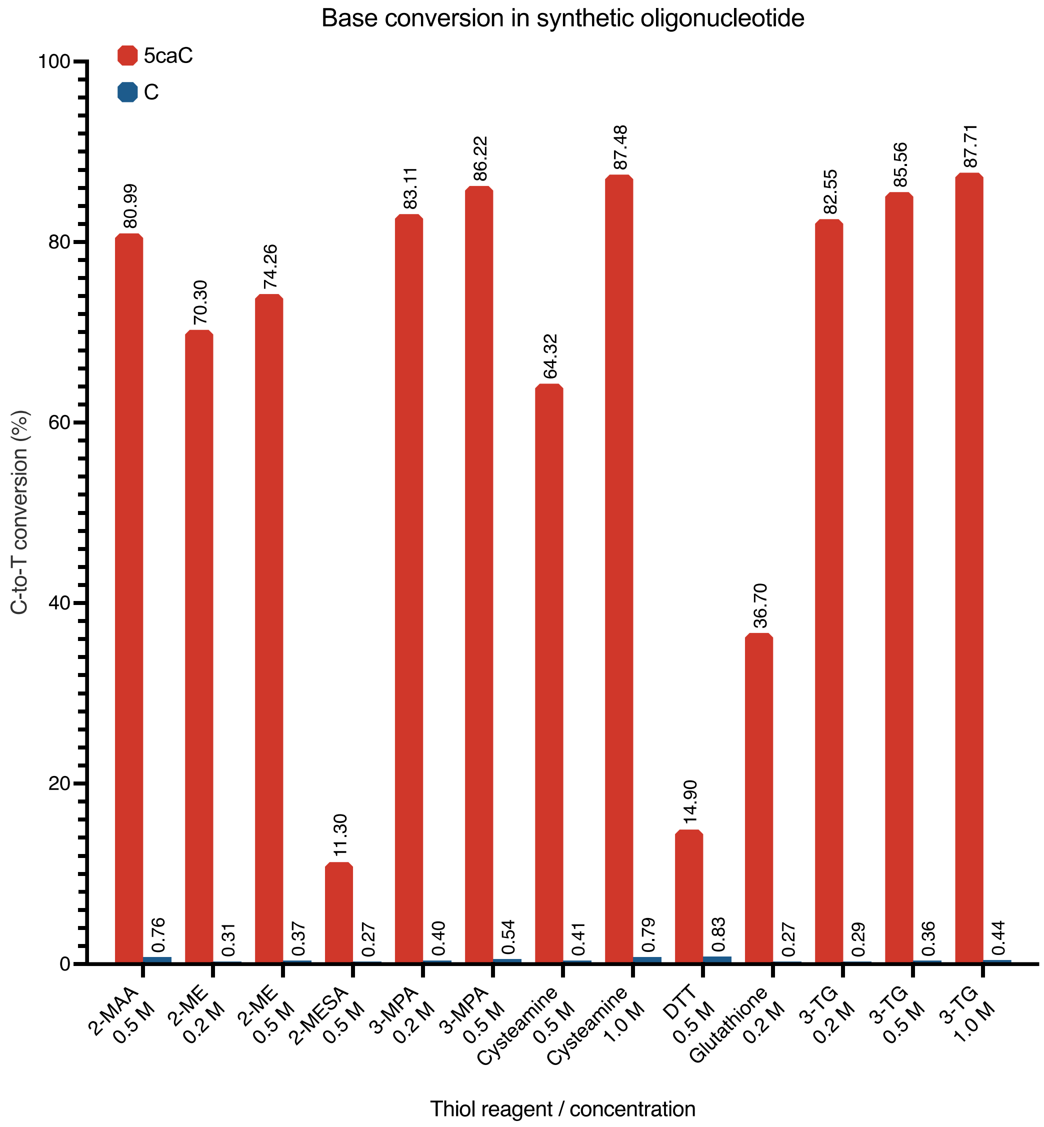

Base conversion outcomes observed by sequencing the synthetic 74mer treated for 10 minutes with different thiol reagents/concentrations. 2-MAA = 2-mercaptoacetic acid; 2-ME = 2-mercaptoethanol; 2-MESA = 2-mercaptosulfonic acid; 3-MPA = 3-mercaptopropionic acid; DTT = 1,4-dithiothreitol; 3-TG = 3-thioglycerol.

Figure S23

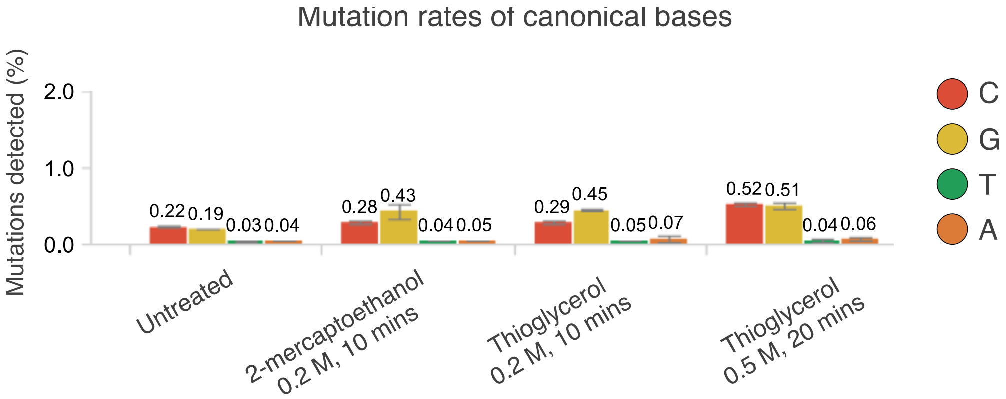

Mutation rates (%) of canonical bases in the modified single-stranded oligonucleotide following different chemical treatments (accompanying Figure 5C; mean of n = 3 ± s.d.).The baseline C-to-T conversions observed in the untreated control samples are the product of errors during PCR amplification and sequencing, as well as nucleoside misincorporation during oligonucleotide synthesis.

λ-Phage DNA

Table S5: CpG modification states in λ-phage genomic DNA

Base	C	5mC	5hmC	5fC	5caC
% of CpG sites	26.9 ± 1.0	n.d.	8.34 ± 0.2	8.22 ± 0.6	56.54 ± 0.5

Abundances of cytosine modifications measured in M.SssI/TET-treated λ-phage genomic DNA, as determined by LC-MS analysis (mean of n = 3 measurements ± std deviation).

Table S6: Description of sequencing libraries prepared from λ-phage DNA

3-Thioglycerol was used as the thiol unless otherwise specified. Reactions were performed on 100 ng input of adapter-ligated DNA, using 0.1 mM photocatalyst and 20% v/v MeCN.

Entry	Thiol concentration (M)	Buffer concentration (mM)	Illumination period (minutes)	Notes	Calculated 5caC conversion efficiency (%) ± s.d.	Off-target conversion of unmodified cytosines (%)
1	0.2	100	20		51.9 ± 2.6 (n = 4)	0.19
2	0.2	50	20		59.8 ± 0.3 (n = 2)	0.25
3	0.2	50	20	16h dark hydrolysis	43.3 ± 4.8 (n = 2)	0.24
4	0.2	50	30	2h dark hydrolysis	71.7 ± 0.1 (n = 2)	0.37
5	0.5	100	20		54.8 ± 3.3 (n = 3)	0.32
6	0.5	50	20		66.1 ± 1.4 (n = 2)	0.33
7	0.5	50	20	16h dark hydrolysis	82.6 ± 7.5 (n = 2)	0.43
**8**	**0.5**	**25**	**20**	**2h dark hydrolysis**	**85.0 ± 1.8 (n = 2)**	**0.38**
9	0.5	250	20		41.4 ± 0.6 (n = 2)	0.20
10	0.5	100	20	*2-mercaptoethanol*	50.2 (n = 1)	0.24
11	1.0	100	20	*2-mercaptoethanol*	44.6 (n = 1)	0.32
12	0.5	100	20	*3-mercaptopropionic acid*	82.9 (n = 1)	0.60

3E – NMR spectra

Figure S24 - ^{1}H NMR & ^{13}C NMR (D_2O) of 2'-deoxy-5-carboxycytidine

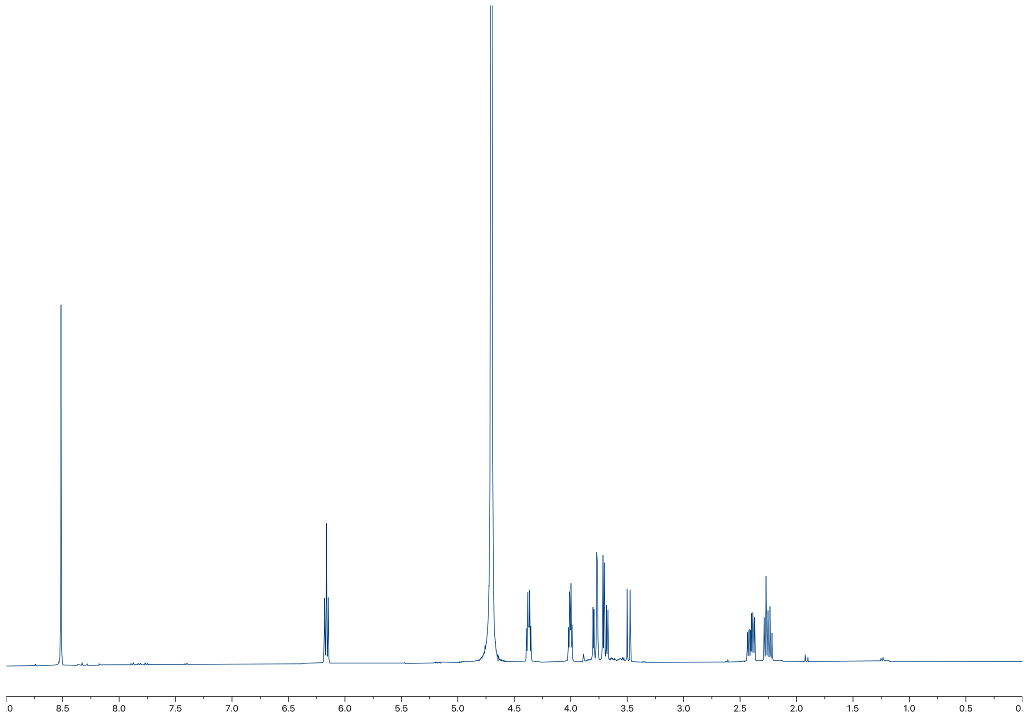

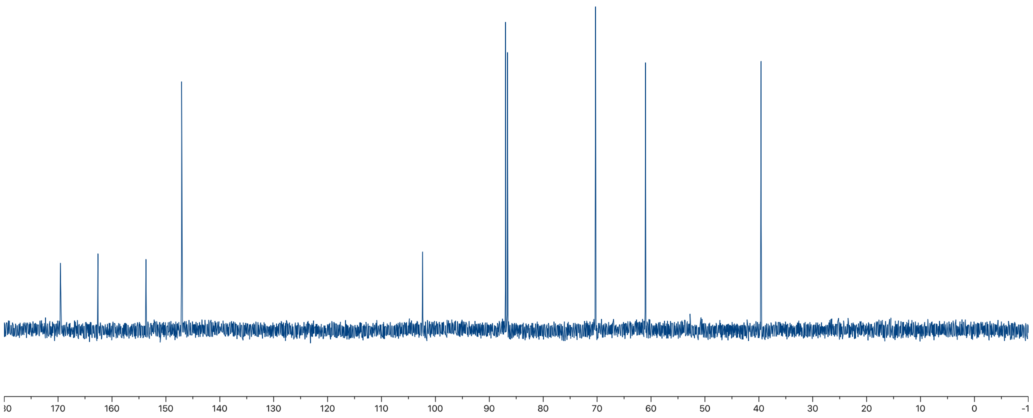

Figure S25 - ^{1}H NMR & ^{13}C NMR (D_2O) of 2'-deoxy-5-carboxymethylcytidine

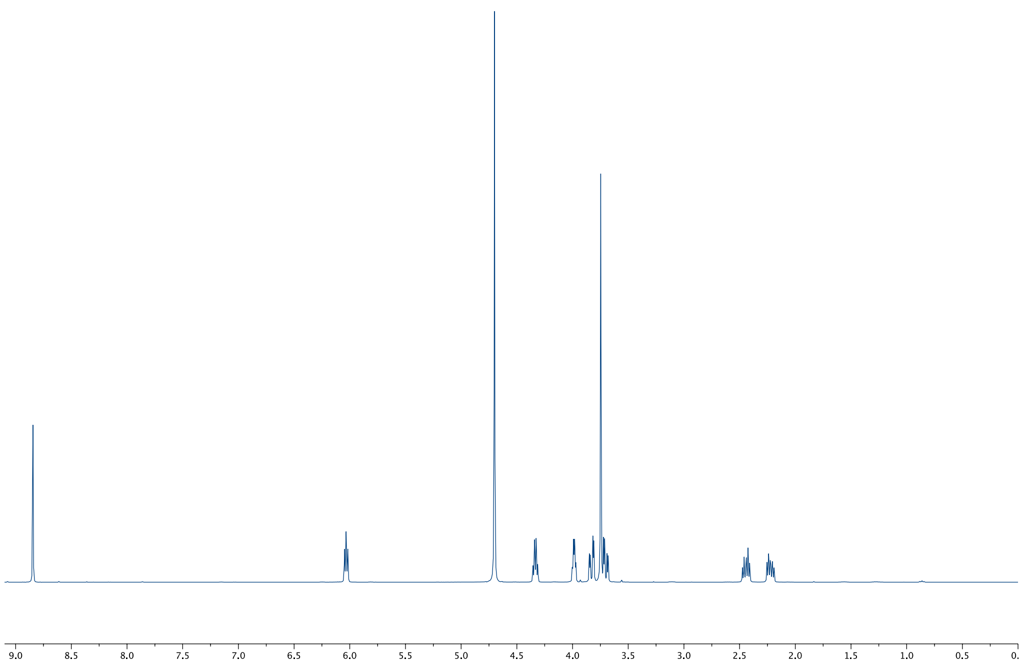

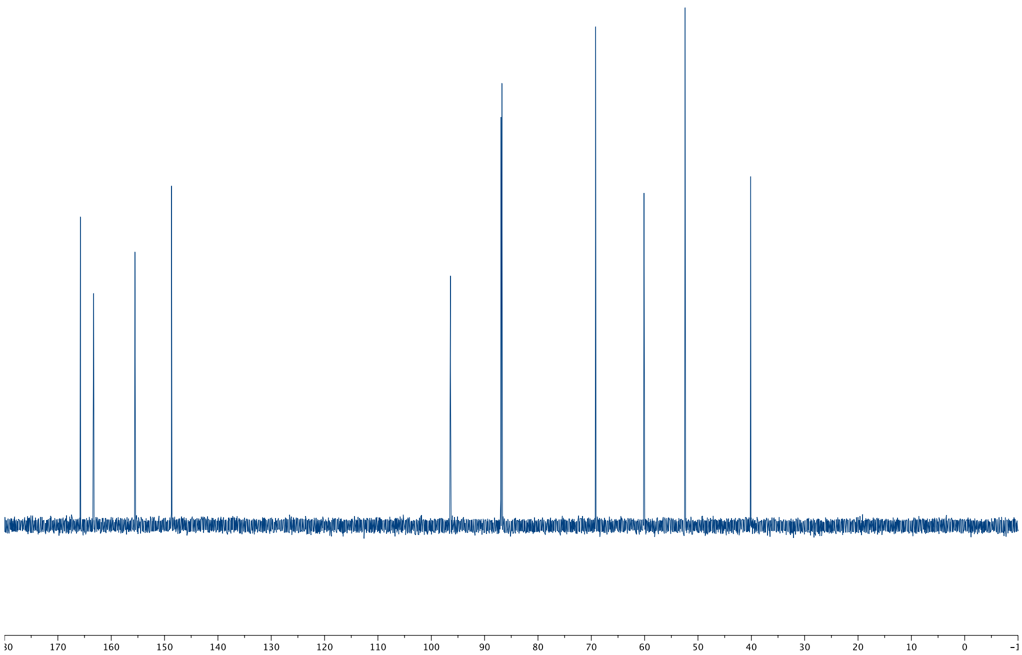

Figure S26 - ^{1}H NMR & ^{13}C NMR (D_2O) of 2'-deoxy-5,6-dihydrouracil

Superimposed on spectra obtained from a commercial sample of DHU (blue)

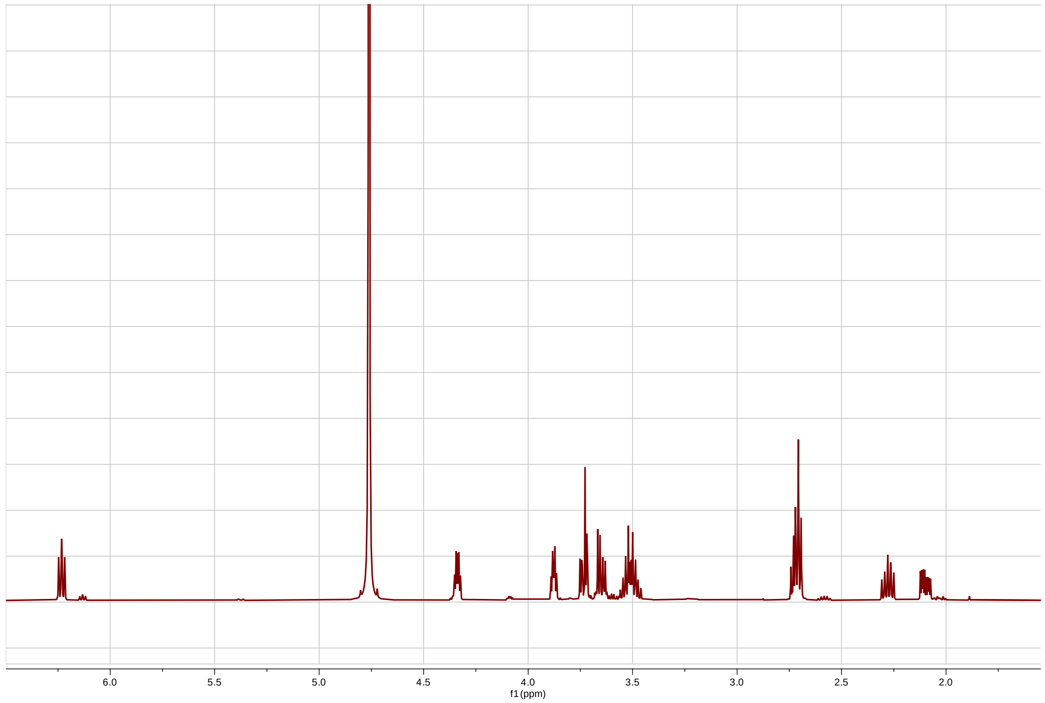

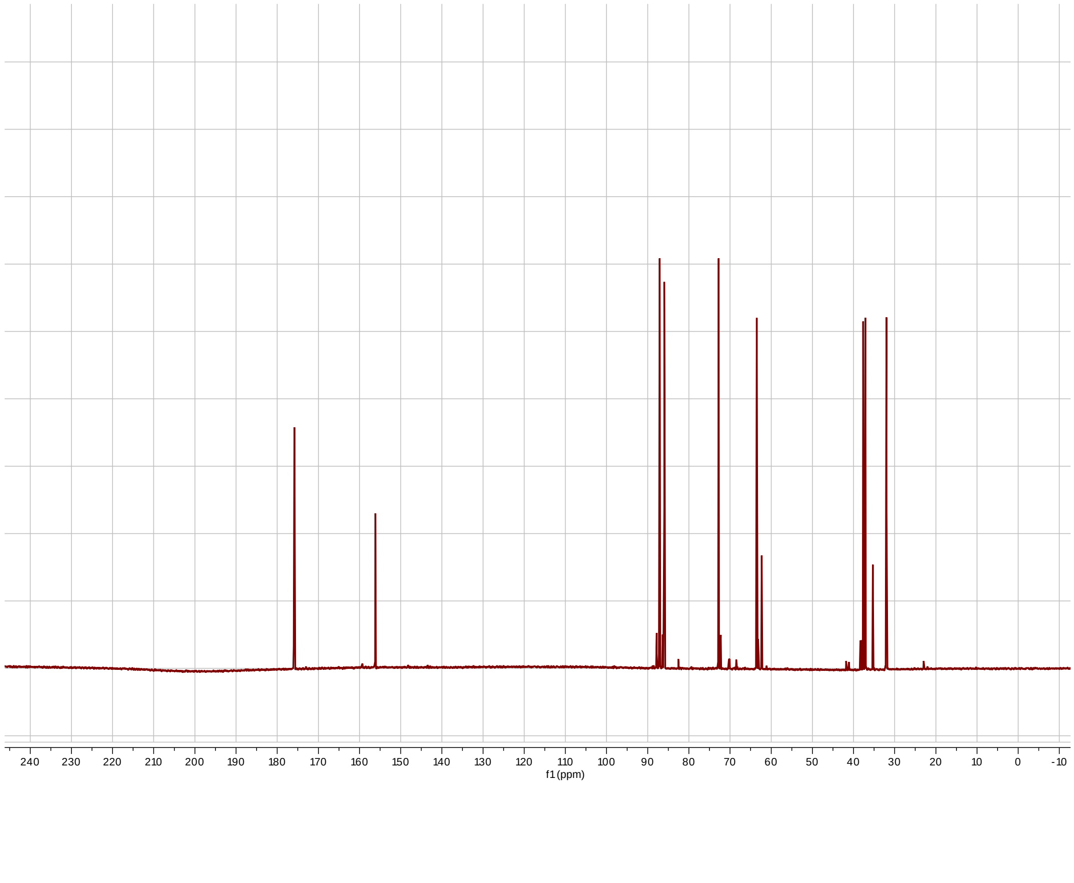

References

(1) Schiesser, S.; Pfaffeneder, T.; Sadeghian, K.; Hackner, B.; Steigenberger, B.; Schröder, A. S.; Steinbacher, J.; Kashiwazaki, G.; Höfner, G.; Wanner, K. T.; Ochsenfeld, C.; Carell, T. Deamination, Oxidation, and C–C Bond Cleavage Reactivity of 5-Hydroxymethylcytosine, 5-Formylcytosine, and 5-Carboxycytosine. *J. Am. Chem. Soc.* **2013**, *135* (39), 14593–14599. https://doi.org/10.1021/ja403229y.

(2) Schröder, A. S.; Kotljarova, O.; Parsa, E.; Iwan, K.; Raddaoui, N.; Carell, T. Synthesis of (*R*)-Configured 2′-Fluorinated MC, HmC, FC, and CaC Phosphoramidites and Oligonucleotides. *Org. Lett.* **2016**, *18* (17), 4368–4371. https://doi.org/10.1021/acs.orglett.6b02110.

(3) Liu, Y.; Siejka-Zielińska, P.; Velikova, G.; Bi, Y.; Yuan, F.; Tomkova, M.; Bai, C.; Chen, L.; Schuster-Böckler, B.; Song, C.-X. Bisulfite-Free Direct Detection of 5-Methylcytosine and 5-Hydroxymethylcytosine at Base Resolution. *Nat. Biotechnol.* **2019**, *37* (4), 424–429. https://doi.org/10.1038/s41587-019-0041-2.

(4) Neese, F. The ORCA Program System. *WIREs Comput. Mol. Sci.* **2012**, *2* (1), 73–78. https://doi.org/10.1002/wcms.81.

(5) Weigend, F.; Ahlrichs, R. Balanced Basis Sets of Split Valence, Triple Zeta Valence and Quadruple Zeta Valence Quality for H to Rn: Design and Assessment of Accuracy. *Phys. Chem. Chem. Phys.* **2005**, *7* (18), 3297. https://doi.org/10.1039/b508541a.

(6) Weigend, F. Accurate Coulomb-Fitting Basis Sets for H to Rn. *Phys. Chem. Chem. Phys.* **2006**, *8* (9), 1057. https://doi.org/10.1039/b515623h.

(7) Goerigk, L.; Grimme, S. A Thorough Benchmark of Density Functional Methods for General Main Group Thermochemistry, Kinetics, and Noncovalent Interactions. *Phys. Chem. Chem. Phys.* **2011**, *13* (14), 6670. https://doi.org/10.1039/c0cp02984j.

(8) Roy, L. E.; Jakubikova, E.; Guthrie, M. G.; Batista, E. R. Calculation of One-Electron Redox Potentials Revisited. Is It Possible to Calculate Accurate Potentials with Density Functional Methods? *J. Phys. Chem. A* **2009**, *113* (24), 6745–6750. https://doi.org/10.1021/jp811388w.

(9) Barone, V.; Cossi, M. Quantum Calculation of Molecular Energies and Energy Gradients in Solution by a Conductor Solvent Model. *J. Phys. Chem. A* **1998**, *102* (11), 1995–2001. https://doi.org/10.1021/jp9716997.

(10) Roth, H.; Romero, N.; Nicewicz, D. Experimental and Calculated Electrochemical Potentials of Common Organic Molecules for Applications to Single-Electron Redox Chemistry. *Synlett* **2015**, *27* (05), 714–723. https://doi.org/10.1055/s-0035-1561297.

(11) Seidel, C. A. M.; Schulz, A.; Sauer, M. H. M. Nucleobase-Specific Quenching of Fluorescent Dyes. 1. Nucleobase One-Electron Redox Potentials and Their Correlation with Static and Dynamic Quenching Efficiencies. *J. Phys. Chem.* **1996**, *100* (13), 5541–5553. https://doi.org/10.1021/jp951507c.

(12) Allouche, A.-R. Gabedit-A Graphical User Interface for Computational Chemistry Softwares. *J. Comput. Chem.* **2011**, *32* (1), 174–182. https://doi.org/10.1002/jcc.21600.

(13) Parr, R. G.; Yang, W. Density Functional Approach to the Frontier-Electron Theory of Chemical Reactivity. *J. Am. Chem. Soc.* **1984**, *106* (14), 4049–4050. https://doi.org/10.1021/ja00326a036.
